# The stability of transient relationships

**DOI:** 10.1038/s41598-023-32206-2

**Published:** 2023-04-14

**Authors:** Valentín Vergara Hidd, Eduardo López, Simone Centellegher, Sam G. B. Roberts, Bruno Lepri, Robin I. M. Dunbar

**Affiliations:** 1 Computational and Data Sciences Department, George Mason University, Fairfax, 22030 USA; 2 Fondazione Bruno Kessler, Mobile and Social Computing Lab, Trento, 38123 Italy; 3 School of Psychology, Liverpool John Moores University, Liverpool, L3 3AF UK; 4 Department of Experimental Psychology, University of Oxford, Oxford, OX2 66G UK

**Keywords:** Complex networks, Computational science, Applied physics

## Abstract

In contrast to long-term relationships, far less is known about the temporal evolution of transient relationships, although these constitute a substantial fraction of people’s communication networks. Previous literature suggests that ratings of relationship emotional intensity decay gradually until the relationship ends. Using mobile phone data from three countries (US, UK, and Italy), we demonstrate that the volume of communication between ego and its transient alters does not display such a systematic decay, instead showing a lack of any dominant trends. This means that the communication volume of egos to groups of similar transient alters is stable. We show that alters with longer lifetimes in ego’s network receive more calls, with the lifetime of the relationship being predictable from call volume within the first few weeks of first contact. This is observed across all three countries, which include samples of egos at different life stages. The relation between early call volume and lifetime is consistent with the suggestion that individuals initially engage with a new alter so as to evaluate their potential as a tie in terms of homophily.

## Introduction

Humans are social animals and having strong and supportive relationships with others has large effects on both physical and mental health^1,2^. These social relationships are not static, but change over time due to two key processes. First, relationships have a natural tendency to weaken over time- to ‘decay’^3^. Indeed, if no effort is made to maintain relationships, the level of emotional closeness between two individuals will tend to decrease^4^ and the relationship will eventually drop out of the person’s social network in terms of the meaningful ties they maintain with others^3^. Long-term studies of people’s social networks (the set of relationships they maintain with their family and friends) show a degree of turnover in network members (alters), with some alters leaving the network and others joining^5,6^. Second, specific life events such as going away to study at university^4,7^, entering a romantic relationship^8–10^, having children^11,12^, or getting divorced^13^ can have an impact on the composition of social networks due to a decrease in the time available to maintain these relationships, or a change in the focus of attention (e.g. making new friends at university). What these various mechanisms highlight is that, despite the need and utility of stable relationships, over a period of time many relationships will cease to be active, i.e. many relationships are *transient* (e.g. Wellman finds that over a 10-year period, only about 27% of relationships remain active in Canadian adults^6^; see also^14^ for a qualitative discussion).

Regardless of causal mechanisms, transient relationships form a considerable fraction of people’s communication (see our results). They are also ubiquitous, judging by the number of studies that report them even when the research is focused on other types of relationships^3–7,11–13,15^. It is easy to appreciate that without them, adapting to the changing social needs of an individual would be impossible as this adaptation involves alters entering and leaving egos’ social networks^15^. However, in contrast to long-term relationships, we know little about the amount of support they provide to a person, how many of them become long-term relations, or whether different individuals can handle more or less of them simultaneously. In summary, we do not have a good understanding of transient relationships as part of dynamic ego social networks.

From a theoretical standpoint, the existing literature does not readily offer a clear picture or definition of transient relationships (only transient romantic relationships seemed to have received systematic attention^16^). On the one hand, the literature on relationship decay has identified a gradual decline in emotional intensity before the end of a relationship^3,4^, suggesting that transient relationships may display a gradually decaying volume of communication. On the other, some research suggests that communication is set to an amount appropriate to the perceived quality of relationships, with longer-lasting relationships receiving a greater amount of communication^6,17,18^. In addition, the literature on homophily and friendship implies that an early and relatively fast assessment of relationships needs to take place in setting such a communication amount^18–21^. Under this picture, the volume of communication gauges the importance of a relationship^15,17^ and the likelihood of the relationship ceasing after some *lifetime*. Thus, whilst research on emotional intensity indicates that relationships are constantly and gradually degrading in the absence of active maintenance, other research on homophily and patterns of communication suggests a rapid evaluation followed by a pattern of steady communication. Further complicating the situation, there is some evidence that emotional intensity and communication volume are monotonically related (see e.g.^7,15^). This begs the question: *are these pictures consistent or contradictory?*

Here, we study a variety of communication data sets, focusing on ties where measured communication is observed to cease, thus signaling a possible relationship hiatus or end. We call these relationships *transient* because their communication is discontinued for a significant amount of time, perhaps permanently. As we show, transient relationships are not just a vanishing component of communication: in all our data sets, a substantial portion of phone calls is invested in transient relationships. By organizing the transient relationships of each ego into groups of similar lifetimes (actively communicating with ego for similar lengths of time), we find that egos display no dominant trends in their communication to such groups of alters; as a group, communication remains steady. This effect is present regardless of lifetime group. Such lack of trend in communication is in marked contrast with the steady decay that the literature reports for the temporal evolution of subjective measures of relationship intensity such as emotional closeness^4^. However, this effect requires lifetimes to exceed a minimum threshold that we characterize and measure. We also find that the call volume an ego invests in a transient tie during the initial weeks of relationship is an informative quantity in estimating tie lifetime. Our results are remarkably robust across cohorts in different countries, of various age ranges, and under different life circumstances. Beyond providing empirical understanding about an important and overlooked class of social relationships, our study suggests that a full understanding of transient relationships requires collecting both objective (e.g. contact events) and subjective (e.g. emotional score) measures of relationship intensity.

Previous research on ego communication patterns has focused on a variety of related questions to the ones asked here such as overall properties of persistence and turnover in communication^15,22^, phone communication survival with individual alters^23,24^, or link prediction in broader communication contexts^25,26^. Whilst this research has provided new insights into both the patterns and dynamics of social relationships, it has not offered specific information on the temporal regularities of communication to individual alters, particularly transient ones.

Although in many areas the study of dynamic networks has gained considerable traction^27–29^, analytical convenience has meant that many studies in the psychology literature on network structure treat relationships as if they are stable over time. Yet, in fact, they are intrinsically dynamic^6,15^. This dynamic property arises partly as a result of changing friendship opportunities and partly as a result of adjustments that people make over time in the value they place on individual relationships. Constraints on the availability of social time result in networks having a layered structure^30–32^ between which individual alters are moved by increases or decreases in the time invested in them, including cases where communication virtually ceases leading to the effective removal of the alter from the layers. Understanding the processes involved in these decisions requires a better appreciation of the communication patterns involved.

It is important to note that the steadiness pattern we uncover here is not incompatible with the well-known burstiness of human communication^33^. Instead, while burstiness indeed plays a role, especially at short time scales when the contrast between activity or inactivity is clearly demarcated, at longer temporal scales such burstiness leads to overall activity levels that can have their own long term patterns such as seasonality and trends. In this study, we are interested in this longer time scale.

Before moving on to the body of the article, we summarize how our findings contrast with the possible hypotheses that the current theory on relationship subjective decay suggests about transient relationships. First, we find no gradual diminishing calling pattern trend. Second, the cessation of relationship communication is not generally presaged by reaching some low level of communication but, instead, is predicted by the volume of communication in the early periods of a relationship. Therefore, our results indicate that the view of transient relationships suggested by the literature on relationship emotional decay is incomplete.

Thus, the key aims of this study are to characterize the temporal communication patterns of transient alters, identify key variables and relations between those, and examine whether these patterns are consistent across different cohorts. We use three different mobile phone call data sets from the US, UK, and Italy, which include people of different ages, life stages, and cultural backgrounds. These data are from the time smartphones were not widely available in the respective countries and therefore do not suffer from the communication channel fragmentation of more recent data, where extensive use of multiple messaging services makes it more difficult to build up a complete picture of an ego’s communication pattern to alters^34,35^. As a parenthetical note, the remainder is exclusively concerned with transient relationships, but we occasionally simply call them relationships for brevity.

## Results

Consider an ego $i\in \mathscr {E}$, where $\mathscr {E}$ is one of the cohorts we study (a data set or subset thereof). The set of alters of *i* is denoted $\mathscr {A}_i$. To develop a clear picture of how an ego-alter relationship evolves over time, we focus on two quantities: the first is the *observed lifetime* $\ell _{i,x}$ of the relationship, i.e. the number of days, reduced by 1, alter $x\in \mathscr {A}_i$ remained in ego *i*’s network from their first until their last observed phone call. The second is the *observed elapsed duration* $a_{i,x}$ of the relationship at the time of a phone call, i.e. the number of days between the first and a subsequent call between *i* and *x*, where the first call is defined to occur at $a_{ix}=0$. By definition, $0\le a_{i,x}\le \ell _{i,x}$. To refer generically to the elapsed duration and observed lifetime of relationships without specifying the ego-alter pair, we simply use *a* and $\ell$ without subindices. For ease of reference, the symbols with their definitions and terms used in this paper are summarized in Table 1.

**Table 1:** All symbols used in this paper, both in the main text and SI. Next to each symbol, there is a brief explanation.

Symbol	Concept	Definition
$\mathscr {E}$	Cohort	Cohort $\mathscr {E}$ which can be US, UK, IT, and IT$_n$.
$\mathscr {A}_i(\ell ,\Delta \ell )$	–	Set of alters of ego *i* with lifetimes between $\ell$ and $\ell +\Delta \ell$.
$t^{c}_{i,x}$	–	Day of *c*th call from ego *i* to alter *x* counted from the start of the data set that *i* and *x* belong to.
$n_{i,x}$	–	Total calls from ego *i* to alter *x*
$a_{i,x}$	Elapsed duration	Observed elapsed duration in days of the relationship between ego *i* and alter *x*.
$\ell _{i,x}$	Lifetime	Observed lifetime in days of alter *x* in ego *i*’s network.
$\Delta t_{s}$	–	Exclusion days at the start of IT data to create IT$_n$. If ego calls alter for the first time at or after $\Delta t_{s}$ days, we identify the relationship as *new*.
$\Delta t_{w}$	–	Exclusion days before the end of the cohort data. If the last contact between an ego-alter pairs occurs $\Delta t_{w}$ days or more before the end of data in their cohort, we identify a relationship as *transient*.
$f_{i,x}(a_{ix},\ell _{ix})$	–	Volume of communication, measured as number of phone calls from ego *i* to alter *x* at elapsed duration $a_{ix}$ of their relationship of lifetime $\ell _{ix}$.
$\Delta a$	–	Time window within which to measure call volume.
$\Delta \ell$	–	Time window for the selection of relationship lifetimes.
$\bar{f_{i}}(a, \ell )$	–	Average per alter number of phone calls from ego *i* to its alters with lifetime between $\ell$ and $\ell +\Delta \ell$ at elapsed duration between *a* and $a+\Delta a$.
$\bar{f}(a, \ell )$	–	Average of $\bar{f_{i}}(a, \ell )$ over all egos.
$b(\ell )$	–	Steady volume of communication to alters with lifetime between $\ell$ and $\ell +\Delta \ell$.
$p_i$	–	*p*-value of the Kolmogorov-Smirnov test between the first and second half of $\bar{f}_i(a,\ell )$.
$g_{i,x}(a_{o}, a_{f})$	–	Number of phone calls from ego *i* to alter *x* when the relationship is between elapsed durations $a_{o}$ and $a_{f}$.
$P(a \mid a_{o}, a_{f}, \gamma )$	Survival probability	Probability that an alter is active at elapsed duration *a*, given that it had activity $3^{\gamma }\le g<3^{\gamma +1}$ during the interval $[a_{o}, a_{f}]$
$I(\ell , g)$	Mutual information	Mutual information between $\ell$ and *g*. It quantifies the amount of information that can be obtained from one variable by observing the other.
$U(\ell , g)$	Symmetric uncertainty	Symmetric uncertainty between $\ell$ and *g*. It measures the same as $I(\ell , g)$, but in a scale that goes from 0 (when the variables are independent) to 1 (when the information one variable gives about the other is complete).

Since we are interested in studying relationships in which contact stops for a sufficiently long time that one can assume that the communication has either ceased or become dormant, in all our cohorts we eliminate from consideration ego-alter pairs that have contact with each other within a time window of $\Delta t_{w}$ days before the last day $T_{\mathscr {E}}$ of data for cohort $\mathscr {E}$; the larger $\Delta t_{w}$, the more stringent our filter is in terms of which relationships we select as having ceased. Note that many relationships that cease communication may be dormant for a considerably longer time than $\Delta t_w$, as they may stop communication well before the end of a data set approaches; $\Delta t_w$ is therefore a lower cutoff of the duration of time without contact (over all our datasets, on average transient relationships cease communication 238 days before the end of their studies). Our method follows a similar logic to^23,24^, and although a small percentage of relationships could become active again as indicated in these references (3% after 6 months), the level of error this induces is very small. Note that, since longitudinal data is always limited, other criteria to determine tie end is very difficult, or even impossible, to apply. Finally, for each cohort $\mathscr {E}$, we limit the relationship lifetimes we study to a maximum value $\mathscr {L}_{\mathscr {E}}$ to avoid issues of poor sampling (see details in Supplementary Information, Sect. S1). These filters lead to three cohorts for the UK, Italy, and the US (see Table 2); the Italian cohort is filtered one more time for additional analysis (so called IT${}_n$ subcohort, see “Data” section below, as well as Supplementary Information, Sect. S1.4.2).

To provide a sense for the magnitude of communication volume to transient alters, we note that for $\Delta t_w=60$ days, each cohort exhibits large proportions of activity dedicated to transient relations. For ties that involved more than just casual exchanges (defined here as at least 3 calls): (i) in the UK cohort they take up ~ 45% of overall communication, (ii) in the US cohort they receive ~ 27% of overall communication, and (iii) in the Italy cohort they take up ~ 17% of overall communication.

### Stable volume of calls

In order to study the evolution of attention allocation from ego to its alters, we focus on call volume as a function of the elapsed duration and observed lifetime of relationships. Specifically, we measure for each ego *i* the quantity $\bar{f}_{i}(a,\ell )$, namely the per alter average number of phone calls to alters whose lifetimes fall within $\ell$ and $\ell +\Delta \ell$ when the elapsed duration of the relationship is between *a* and $a+\Delta a$ (for definitions of $\Delta a,\Delta \ell$, see “Methods” section). If communication volume exhibits any general trend over the duration of ego-alter relations, $\bar{f}_i(a,\ell )$ would reflect such trend (in the Supplementary Information, Sect. 3.7, we show that another possible way to measure communication, time spent talking, is highly correlated with the number of calls).

To aid in our study of $\bar{f}_i(a,\ell )$, and because any single ego *i* has few alters with a given combination $a,\ell$, we also measure $\bar{f}(a,\ell )$, the average of $\bar{f}_i(a,\ell )$ over egos with $a,\ell$ (using the same $\Delta a$ and $\Delta \ell$ as $\bar{f}_i(a,\ell )$). Intuitively, $\bar{f}_{i}$ and $\bar{f}$ capture stable estimates of the communication volume (attention allocation) egos invest per alter.

We first focus on the UK cohort (as described in greater detail in Materials and Methods and Supplementary Information, Sect. S1) which is extracted from a study of students in their last months of secondary school and their first entire year of university study^36^. From this study, we form a cohort comprised of the transient relationships that egos form with alters after they transition to university (6 months from the start of the study)^4^, and that also satisfies the transient relationship filter explained above.

**Figure 1:**
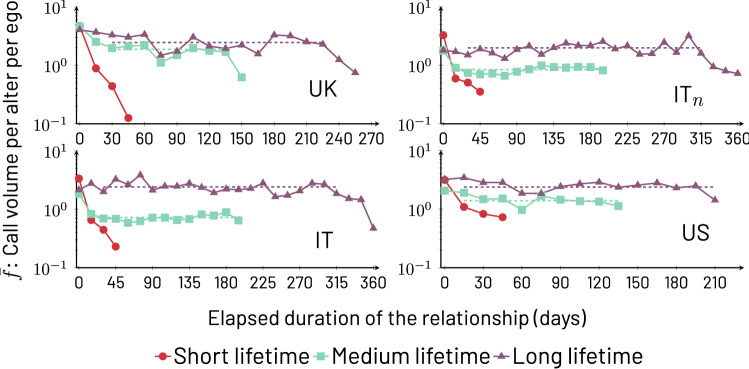
Average per alter per ego phone call volume $\bar{f}(a,\ell )$ as a function of elapsed relationship duration *a*, binned with $\Delta a=15$ and $\Delta \ell =50$ for the four cohorts shown. The lifetime groups correspond to $\ell =0$ (short), $\ell =\lfloor (\mathscr {L}_{\mathscr {E}}-\Delta \ell )/2\rfloor$ (medium), and $\ell =\mathscr {L}_{\mathscr {E}}-\Delta \ell$ (long). To calculate the exact $\ell$ per country, as stated in Fig. S1 of the Supplementary Information, we use $\mathscr {L}_{\textrm{UK}} = 270$; $\mathscr {L}_{\textrm{IT}_{n}} = 365$; $\mathscr {L}_{\textrm{IT}} = 365$; and $\mathscr {L}_{\textrm{US}} = 220$. The transient condition is $\Delta t_w=60$ days, and for cohort IT${}_n$, the gap between the entry of an ego and the acceptance of an ego-alter pair is set to $\Delta t_s=50$ days. The number of resulting ego-alter pairs induced by our selection criteria is reported in Table 2. Robustness checks with different values for parameters $\Delta \ell , \Delta a,\Delta t_w$, and $\Delta t_s$ are shown in the Supplementary Information, Sect. S3. The curves are stable for medium and long lifetime groups. For curves displaying stable regions, we show a dashed line that represents $b(\ell )$, the average number of phone calls to alters of a given $\ell$ during the stable regime of communication.

**Figure 2:**
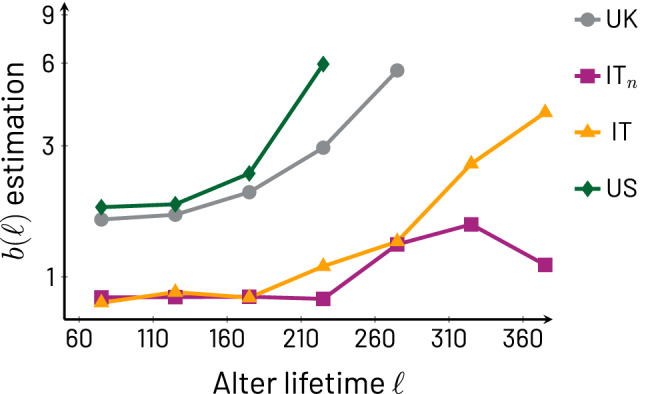
$b(\ell )$ as a function of $\ell$ obtained through the stable region average method (see Methods section). The vertical axis is in logarithmic scale. Clearly, $b(\ell )$ has an increasing trend with respect to $\ell$, with minor exceptions. The UK and US cohorts display a faster increase than IT and IT${}_n$. This could be a consequence of specific differences between details of the cohort participants, such as country, age, and/or personal circumstances of the participants; for example, since the Italian cohort is focused on adult parents with pre-teenage children, these participants may have less available time to invest in phone communication.

The new alters that emerge after 6 months of the start of the study are almost certainly new social relationships for the egos, as prior research has shown that almost no relationships survive after 6 months without communication^18,23^. In this cohort, *a* and $\ell$, respectively, approximate very well the *actual* duration and lifetime of transient relationships. In Fig. 1 (UK), we present $\bar{f}(a,\ell )$ for three groups of transient relationships based on their lifetimes: short starting with $\ell =0$, medium starting with $\ell =\lfloor (\mathscr {L}_{\mathscr {E}}-\Delta \ell )/2\rfloor$, and long starting with $\ell =\mathscr {L}_{\mathscr {E}}-\Delta \ell$; in all cases, $\Delta \ell =50$, and $\lfloor \rfloor$ represents the floor function. We standardize these ranges for this and subsequent analysis of $\bar{f}(a,\ell )$ and $\bar{f}_i(a,\ell )$ to avoid idiosyncratic choices, but see our comments about lifetime ranges in the discussion of Fig. 2. First, we note that alters with longer lifetimes receive a greater volume of calls (i.e. $\bar{f}(a,\ell _1)>\bar{f}(a,\ell _2)$ if $\ell _1>\ell _2$). Second, lifetime groups exhibit an initial period of slightly elevated activity up to an elapsed duration we label $a_s$ and, after this period, medium and long lifetime groups exhibit $\bar{f}(a,\ell )$ that stabilize with respect to *a*, remaining close to constant for a long range of values of *a*, or

1 $$\begin{aligned} \bar{f}(a,\ell )\approx b(\ell )\quad [a_{s} \lesssim a \lesssim \ell ; \ell \gtrsim \ell _s], \end{aligned}$$

where $\ell _s$ is the value of lifetime when the steady behavior sets in (see below). In other words, for $\ell >\ell _s$, $\bar{f}(a,\ell )$ approaches an *a*-independent value $b(\ell )$ from about $a_s$ (which corresponds to a value of 3 days, as described in the Supplementary Information Fig. S8) to just before the observed lifetime ($a \gtrsim \ell$). Both $b(\ell )$ and $\ell _s$ are determined by finding the range of *a* where, respectively, $\bar{f}(a,\ell )$ and $\bar{f}_i(a,\ell )$ become steady. Note that $\ell _s$ marks the upper bound for another type of transient relationship with $\ell <\ell _s$, one that is too short and ephemeral to achieve any stability; in Fig. 1, all short lifetimes correspond to this type. We estimate $\ell _s$ as explained in the Supplementary Information, Sect. S5.4, and find that, depending on the estimation technique, the average value for all the cohorts studied here ranges from $\approx 56$ to 62 (values for individual cohorts are similar, and are reported in Supplementary Information, Table S2). In this study, we do not pursue this line of inquiry further.

**Table 2:** Number of transient ego-alter pairs by cohort with $\Delta t_w=60$ days (and $\Delta t_s=50$ days for IT${}_n$ only). The last three columns show the exact number of relationships specifically used in the lifetime groups of Fig. 1 which represent a subset of all the transient relationships contained in the data.

Cohort	Number of egos	Alters	Short lifetime	Medium lifetime	Long lifetime
UK, IT, and US combined	303	7625	–	–	–
UK	30	920	483	90	76
IT${}_n$	142	2736	1102	278	157
IT	143	4052	1369	447	313
US	130	2653	1415	399	319

The UK cohort, while highly informative because of being constituted almost purely of new relationships (transient or long-lasting), is limited by its size (30 egos) and represents only one example of the behavior shown. To strengthen our results, we introduce the US and Italy data sets, which have a larger number of egos (for details see “Data” section and Supplementary Information, Sect. S1)^37,38^. The Italian data set in particular has both a large sample and a longer duration, allowing us to construct two different analyses to support our findings. Further, whilst the UK cohort was specifically recruited to capture a period of transition in the egos’ social networks^15^, the Italian and US data were collected for egos under steadier social circumstances which could, in principle, lead to different characteristics of transient relationships. Therefore, studying transient relationships across these three cohorts provides a test of the robustness of the findings of trendlessness in communication in transient relationships, using egos at different life stages.

Whilst in the UK, *a* and $\ell$ accurately reflect actual elapsed duration and lifetime of transient relationships, respectively, for the Italian and US studies these measures become approximate as the precise start of a relationship cannot be guaranteed to occur after the study was initiated. In order to provide a second test that transient relationships in other contexts have the behavior observed in the UK, we create a subcohort IT${}_n$ out of the Italian data in which, for an ego, we restrict the ego-alter pairs to those that satisfy both the transient criterion ($\Delta t_w$) *and* begin at least $\Delta t_s$ days after the entry of the ego into the study. Beyond providing a cross-check for the UK results, in this subcohort *a* and $\ell$ accurately reflect actual elapsed duration and lifetime. Figure 1 shows the equivalent analysis of the UK subcohort, now for IT${}_n$, with remarkably consistent results.

As we show next, the robustness of the behavior of transient relations is such that even a more approximate measurement of *a* and $\ell$ continues to be informative. In the two bottom panels of Fig. 1, we present $\bar{f}(a,\ell )$ for both the Italian and US cohorts still restricted to transient relationships but without restricting the timing of the entry of ego-alter pairs. The communication patterns in these cohorts are once again consistent with those of the UK and IT${}_n$. This should not be surprising because, given that one is selecting for transient relationships, the properties they possess lead to the same qualitative patterns (steady $\bar{f}(a,\ell )$ with a growing tendency as a function of $\ell$). The nature of the approximation in using these cohorts is reflected in the measurement of $\ell$, particularly if it is to be interpreted as *actual* lifetime of a relationship. If we define $\hat{\ell }$ and $\ell$ as, respectively, the actual and the observed lifetimes, then the Italian and US cohorts can have examples of $\hat{\ell }>\ell$ for particular relationships, whereas for the UK and IT${}_n$, one expects $\hat{\ell }\approx \ell$. In reality, only a fraction of ego-alter pairs in the unrestricted Italian and US cohorts are affected by this, because many relationships indeed start a considerable amount of time after an ego enters a study (average entry day per cohort: 119 UK, 287 IT${}_n$, 292 IT, and 283 US; complete distributions found in Supplementary Information, Fig. S2). Below, we take advantage of the robustness with respect to the measurement of $\ell$ to perform the analysis leading to Figs. 4 and 5 with the UK, Italy, and US only since they provide larger statistical sampling.

**Figure 3:**
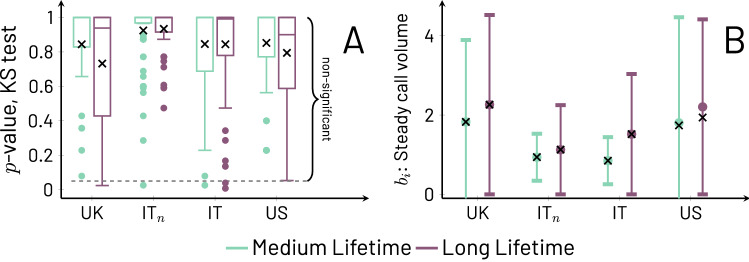
Panel (**A**): Box plots for all cohorts using the 1.5 interquartile range convention for *p*-values from Kolmogorov-Smirnov tests for egos in medium (teal) and long (purple) lifetimes in all cohorts. The per alter call averages $\bar{f}_i(a,\ell )$ are divided into two equally-sized ranges of *a*, the early range $\Delta a\le a< \lfloor (1/2)\left( \lfloor \ell /\Delta a\rfloor -1\right) \rfloor \Delta a$ and the late range $\lfloor (1/2)\left( \lfloor \ell /\Delta a\rfloor -1\right) \rfloor \Delta a\le a\le \lfloor \ell /\Delta a \rfloor \Delta a-\Delta a$). Large *p*-values mean that the early and late ranges of $\bar{f}_i(a,\ell )$ are not distinguishable, and thus, show no trend with *a*; small *p*-values mean there is a trend in *a*. We draw a dashed line at the 0.05 significance threshold and the averages over all egos are represented with the symbol $\times$. As it is clear from the plot, the vast majority of egos show no trend with *a*. Panel (**B**): Average values of $b_i(\ell )$ (circles) and standard error of the means (whiskers) for medium (teal) and long (purple) lifetimes for all cohorts. Superimposed to each circle and associated whisker is a symbol $\times$ that represents the value of $b(\ell )$ for the corresponding cohort, which matches well the averages of $b_i(\ell )$ across cohorts and lifetimes.

As noted above, $b(\ell )$ is observed to increase as a function of $\ell$. To provide further evidence for this observation, we present Fig. 2 which systematically displays this relation. The fact that $b(\ell )$ increases with $\ell$ highlights that our selection of the medium and long lifetimes used in Figs. 1 and 3 (below) does not affect the conclusions we draw about the behavior of $\bar{f}(a,\ell )$; in other words, one can work with values of $\ell$ from $\ell _s$ and up. From Fig. 2 we also note that, while the trends of $b(\ell )$ are increasing, there are differences among the cohorts, with the US and UK showing a more rapid growth than the Italian cohorts, which start roughly steady and then begin their marked increase at larger values of $\ell$ (the IT$_n$ cohort shows one decaying point for the largest $\ell$, due to sampling issues, as discussed in the Supplementary Information, Sect. S3.2). This may have implications in terms of how effectively one can distinguish medium lifetimes in Italian ego-alter pairs in comparison to the other cohorts on the basis of early phone call activity.

While $\bar{f}(a,\ell )$ allows us to describe the temporal patterns of communication more easily, this is an average quantity over egos and therefore may not be representative of $\bar{f}_i(a,\ell )$. However, it is the latter quantity that genuinely interests us because it captures a more accurate picture of how each ego generally behaves with its alters, i.e., what are the trends in communication over time. To examine $\bar{f}_i(a,\ell )$, we carry out two analyses. The first one consists of determining the level of steadiness of $\bar{f}_i(a,\ell )$ as a function of *a*. This is done ego by ego, taking for each time series $\bar{f}_i(a,\ell )$ two parts of equal duration in *a* around the mid-point of the series and excluding the first ($a=0$) and last ($a=\lfloor \ell /\Delta a \rfloor \Delta a$) points (details found in Methods). The two ranges of elapsed duration generate for each ego two samples of $\bar{f}_i(a,\ell )$ at points in *a* within each of the periods, and we perform a Kolmogorov-Smirnov test to determine if the values of the two samples come from the same distribution. Figure 3A captures the results of the test. Overwhelmingly, the test shows non-significant differences between the values of $\bar{f}_i(a,\ell )$ before and after the mid-point, ego by ego. Moreover, the average *p*-values of the tests over each and every $\bar{f}_i(a,\ell )$ are actually quite high (see symbols in Fig. 3A, values range between 0.73 and 0.94, and are reported per cohort and lifetime in Supplementary Information, Fig. S15), not merely rejecting the possibility of change, but confirming a high probability that communication volumes remain largely unchanged between time periods. In other words, $\bar{f}_i(a,\ell )$ remains steady between the first and second periods of the lifetime. The second analysis pertains to the robustness of $b(\ell )$ as a good approximation for $\bar{f}_i(a,\ell )$ or, more precisely, that each individual $\bar{f}_i(a,\ell )$ does not deviate much from $b(\ell )$. We test this by calculating $b_i(\ell )$ for each ego and form its distribution over *i* (see Fig. 3B). The results show that indeed the values of $b_i(\ell )$ are typically close to those of $b(\ell )$ and, therefore, can be treated as approximately equal, i.e. $b_i(\ell )\approx b(\ell )$.

The results illustrated by Figs. 1, 2, and 3 together support the following interpretations. First, the pattern of communication that each ego maintains with its transient alters does not exhibit systematically increasing or decaying trends, that is, no trend is dominant (unless $\ell <\ell _s$, in which case there do not seem to be stable relationships). This steadiness due to the absence of trends is strongly supported by the lack of statistically significant results, and indeed large *p*-values approaching 1, from the Kolmogorov-Smirnov test comparing the first and second time periods of each ego’s call volumes $\bar{f}_i(a,\ell )$. The steadiness is a surprising result that indicates that communication related to transient relationships does not tend to gradually fade away in parallel with measures of emotional closeness ^4^; when communication ceases, it appears to do so without warning. Second, the similarity between $b(\ell )$ and the set of $b_i(\ell )$ (that is, $b_i(\ell )\approx b(\ell )$) shows that the $b_i(\ell )$ follow a growing trend with $\ell$. This trend, displayed in Fig. 2 for $b(\ell )$, also means that the definitions of medium and long lifetimes used in Figs. 1 and 3 can be changed without affecting our conclusions. Third, the fact that the behavior of various cohorts is in agreement means that the variables *a* and $\ell$ capture useful measures of transient relationship duration and lifetime even if the start of a relationship has not always been observed in a study. Fourth, in Fig. 1 a number of curves begin with an elevated volume of communication and rapidly settle to their steady long-lasting behavior.

### Survival of alters

The increase of the $b_i(\ell )$ with respect to $\ell$ suggests that it may be possible to estimate $\ell$ for transient relationships on the basis of the communication volume they maintain. Note that while $b_i(\ell )$ is not specific to a given relationship of ego *i*, it is nevertheless formed by the aggregation of ego *i*’s communication with alters of lifetime $\ell$ and therefore each individual relationship’s communication volume is likely to be of a similar scale as $b_i(\ell )$.

Let us define $g_{i,x}(a_o,a_f)$ as the number of phone calls ego *i* places to alter *x* when their relationship is between observed elapsed durations $a_o$ and $a_f$ ($g_{i,x}$ is an $\ell$-unrestricted version of $f_{i,x}$). The increase of $b_i(\ell )$ with $\ell$ suggests that, for a randomly chosen $x\in \mathscr {A}_i$, $g_{i,x}$ is likely to increase with $\ell$. To confirm this, we define the probability $P(a \mid a_o,a_f,g)$ over a set of egos (cohorts or combinations thereof) and their transient alters with lifetimes $\ge a_o$ that one of those alters, randomly chosen, with call volume *g* within the window $a_o\le a\le a_f$ is still active for elapsed durations $a> a_o$ (note that *a* can be smaller or larger than $a_f$). The intuition of this quantity is that if we take, for example, the number of calls $g(a_o,a_f)$ placed by an ego to one of its alters in a given period of the relationship (when *a* is between $a_o$ and $a_f$), the probability that the relationship will still be active for $a>a_o$ would grow with the number of calls $g(a_o,a_f)$ received by the alter; in other words, the more calls received, the longer the lifetime. The period comprised by $a_o\le a\le a_f$ can be chosen with some level of flexibility, but if it corresponds to an early period in the observation of the relationship (for example, the second complete month of activity), it may provide an early forecast for the lifetime of the relation. Due to the discreteness of the *g* and the finite sample size, we slightly modify the probability we study to include a range of values of *g*, and represent the quantity by $P(a \mid a_o,a_f,\gamma )$, where $\gamma$ characterizes a range of values of *g* (specifically, $\gamma$ is defined as the exponent characterizing the bin $3^\gamma \le g<3^{\gamma +1}$).

**Figure 4:**
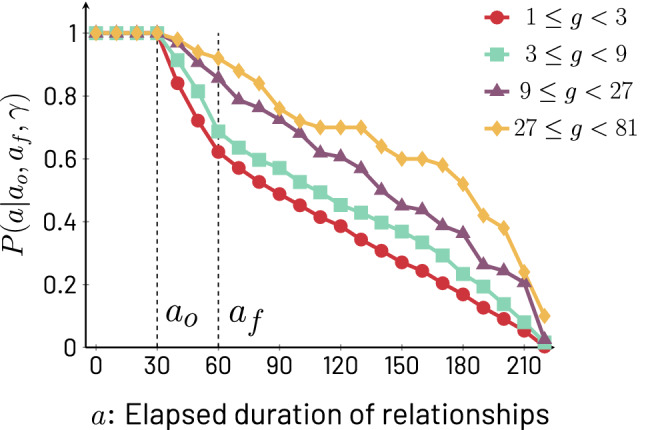
Survival probability $P(a\mid a_o,a_f,\gamma )$ of transient alters to duration of at least *a* for different bins $\gamma$ of amount of mobile phone calls between $a_o=30$ and $a_f=60$ days. We use the combined data for UK, Italy and US, and therefore, we only look at relationships active for $\ell <\mathscr {L}_{\textrm{US}}=220$ days or less, in order to include data for all three cohorts. The bins represented by $\gamma$ as the exponent in $3^{\gamma }\le g<3^{\gamma +1}$ are $\gamma =0,1,2,3$. As $\gamma$ increases, the probability of survival also increases, i.e. for $\gamma '>\gamma$, $P(a\mid a_o,a_f, \gamma ') > P(a\mid a_o,a_f,\gamma )$ which is equivalent to saying that $P(a\mid a_o,a_f,\gamma )$ decays more slowly in terms of *a* as $\gamma$ increases. See Supplementary Information, Fig. S19, for various combinations of $a_o,a_f$.

In Fig. 4, we combine the UK, Italy, and US cohorts to show that there is a monotonically increasing relation between $P(a\mid a_o,a_f,\gamma )$ and $\gamma$, i.e. that the survival probability of a specific alter in an ego’s network grows based on the number of calls ego makes to alter between days $a_o$ and $a_f$ (here taken to be 30 and 60, respectively) of the observed relationship. The monotonic behavior is robust to different choices of parameters and cohorts (see Supplementary Information, Fig. S18). Note that we deliberately used an extremely simple test that captures an early period of relationships, even including the challenging choice of $a_o,a_f<\ell _s$ which means that many alters we consider do not reach steadiness. Nevertheless, the measurement clearly shows the monotonicity of $P(a\mid a_o,a_f,\gamma )$ with $\gamma$. Since this survival analysis is meant to illustrate the relation between $\ell$ and *g*, we refrain from developing this point further, as a more precise prediction of the continuation of relationships may require the use of additional variables beyond call volume. A selection of such variables may be informed by several considerations, including other work that has explored the related (but not identical) question of alter persistence^24^.

### Relation between early call volume and relationship lifetimes

The results displayed in Fig. 4 demonstrate that knowing $g_{i,x}(a_o,a_f)$ for relationship *i*, *x* provides information about $\ell _{i,x}$. Next, we perform two analyses that further illustrate and quantify this.

First, we present the *symmetric uncertainty* $U(\ell , g)$ between the two random variables $\ell$ and *g* measured for each ego-alter pair in each cohort as well as for all unique cohorts combined. This quantity ranges from 0, when $\ell$ and *g* are independent, to 1, when $\ell$ gives complete information on *g* and *vice versa*. Concretely, *U* is a monotonically increasing function of how tightly interdependent two variables are to each other and it is therefore a function of the joint distribution of the variables. Symmetric uncertainty is a normalized version of the more well-known concept of mutual information $I(\ell ,g)$ (see “Methods” section). Combining all cohorts, $U(\ell ,g)=0.09$, while separate cohorts yield $U(\ell , g \mid \textrm{UK}) = 0.3632$, $U(\ell , g \mid \textrm{IT}) = 0.1044$, $U(\ell , g \mid \textrm{IT}_n) = 0.0998$, and $U(\ell , g \mid \textrm{US}) = 0.1597$. Although these values are not near 1, they are nevertheless quite significant, and to interpret them we must take into account that *g* is measured very early in relationships, ignoring other variables related to the value of $\ell$^24^.

The interpretation of Fig. 4, along with the consistency of the results over various cohorts, suggests another interesting possibility: by quantifying the behavior of one cohort, one may be able to predict the behavior of another. In our final analysis, we examine whether $P(a\mid a_o,a_f,\gamma )$ calculated from the combined cohort made of the US and UK data sets can predict the behavior of the Italian cohort.

**Figure 5:**
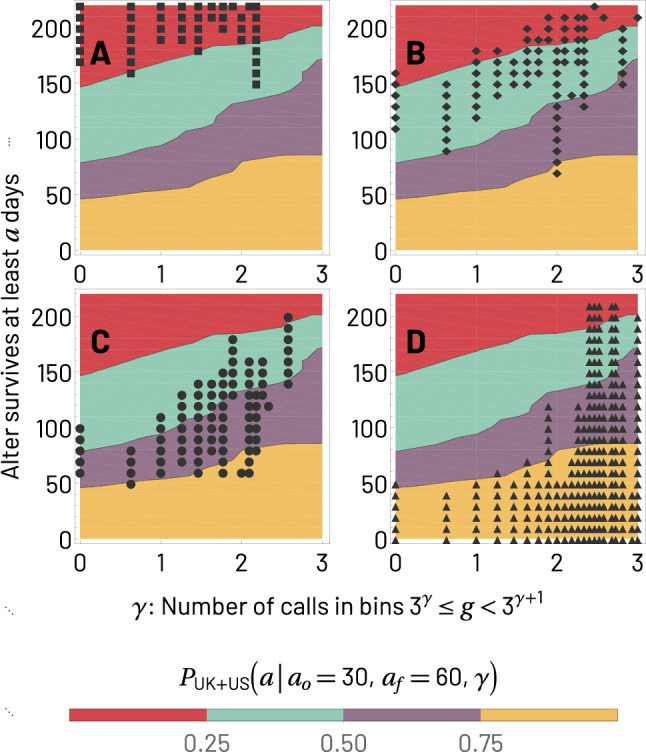
Comparison between the survival probabilities $P(a\mid a_o,a_f,\gamma )$ for the combined UK and US data sets (color map) and the Italian data set (symbols). The different colors of the background represent ranges of $P_{\mathrm{UK + US}}(a\mid a_o,a_f,\gamma )$, namely [0, 0.25) (red), [0.25, 0.5) (teal), [0.5, 0.75) (purple), and [0.75, 1] (yellow). Panel (**A**) shows the symbol $\blacksquare$ for $P_{\textrm{IT}}(a\mid a_o,a_f,\gamma )$ in the interval [0, 0.25), panel (**B**) shows the symbol $\diamond$ for the interval [0.25, 0.5), panel (**C**) uses the symbol $\bullet$ for the interval [0.5, 0.75), and panel (**D**) uses the symbol $\blacktriangle$ for the interval [0.75, 1). The match in location between the symbols and the colored regions means that the behavior of different cohorts is consistent, supporting the reliability of *g* as a helpful predictor of $\ell$.

The results of our analysis are shown in Fig. 5, generated as follows: combining the US and UK cohorts, we calculate $P(a\mid a_o,a_f,\gamma )$ for *a* between 0 and $\mathscr {L}_{\textrm{US}}$ (the smallest value of largest $\ell$ possible among the cohorts in the figure), with the values of the parameters of $a_o,a_f$ and bins $\gamma$ as shown in Fig. 4. Let us call this survival probability $P_{\text {US+UK}}(a\mid a_o,a_f,\gamma )$. The background of Fig. 5 is a 2-dimensional color map version of Fig. 4 with $P_{\text {US+UK}}(a\mid a_o,a_f,\gamma )$, where the horizontal axis captures the call volume in the period between $a_o$ and $a_f$ (here, the second month), the vertical axis captures relationship survival up to duration *a*, and the color represents the value of $P_{\text {US+UK}}(a\mid a_o,a_f,\gamma )$ differentiated into four ranges, [0, 0.25) (red), [0.25, 0.5) (teal), [0.5, 0.75) (purple), and [0.75, 1.0] (yellow). One way to intuitively understand the construction of the color map is to do a parallel transport out of the page of each of the curves in Fig. 4 by an amount proportional to the $\gamma$ associated with call volume between $a_o$ and $a_f$, and then connect the curves along lines of equal probability. These lines of equal probability are the boundaries between colors seen in Fig. 5. To interpret this contour map, note that if we organize the colors in decreasing order of the probability of survival they represent, we obtain the ordered sequence yellow, purple, teal, and red. This order of colors is the same we encounter as we travel the contour map in the direction of increasing *a*, which means that longer lifetimes are less probable. However, note that we can travel along the increasing *a* direction on a variety of parallel paths each corresponding to a fixed value of $\gamma$. Since the lines that separate the colored regions of the contour map bend upwards as $\gamma$ increases, it means that traveling in the increasing *a* direction along a line that has a large fixed $\gamma$, the probability of survival decays more slowly with increasing *a*, indicating that lifetime increases with increased calling in the period between $a_o$ and $a_f$.

To understand the connection between the Italian cohort (represented by the symbols in the panels of Fig. 5) and the combined US and UK cohort (represented by the colored background), we test if the survival probabilities for the two cohorts are similar. Intuitively, we check if $P_{\text {IT}}(a|a_o,a_f,\gamma )$ is similar to $P_{\text {US+UK}}(a|a_o,a_f,\gamma )$ when the two inputs of these functions, the relationship survival time *a* and the volume of early communication $\gamma$, are the same. To test this similarity, we divide the values of $P_{\text {IT}}(a|a_o,a_f,\gamma )$ into the same four ranges used for $P_{\text {US+UK}}(a|a_o,a_f,\gamma )$. Concretely, $P_{\text {IT}}(a|a_o,a_f,\gamma )$ can lay in the range [0, 0.25) (squares), [0, 25, 0.5) (diamonds), [0.5, 0.75) (circles), or [0, 75, 1] (triangles). Now, because *a* and $\gamma$ represent a location in Fig. 5, it means that if the symbols representing a range of $P_{\text {IT}}(a|a_o,a_f,\gamma )$ land in the colored area with corresponding range of $P_{\text {US+UK}}(a|a_o,a_f,\gamma )$, then it means that indeed the probability of survival of alters given a certain amount of early communication volume are similar across the cohorts. For example, if the square symbols land in the red region, it means that the survival probabilities in the range [0, 0.25) for both IT and the combined US and UK cohorts occur for the same survival times and amounts of early activity. Going through the four panels, each corresponding to a different range of values of survival probability, the match in location of $P_{\text {IT}}(a\mid a_o,a_f,\gamma )$ and $P_{\text {US+UK}}(a\mid a_o,a_f,\gamma )$ is clearly visible. There is a small discrepancy between the Italian and combined US and UK cohorts for the long lifetimes at the largest values of $\gamma$, but this effect can be explained from Fig. 2 where we clearly see that given a specific value of volume of communication, lifetimes are longer in Italy than in the US and UK. Notwithstanding this minor discrepancy, the figure shows that indeed the increase in survival time probability of transient relationships for increasing $\gamma$ is a robust phenomenon across countries. In the Supplementary Information, Sect. S7, we construct alternative combinations of countries and find similar consistency.

## Discussion

In this study, we use three mobile phone data sets from the UK, US, and Italy to examine the temporal evolution of communication between an ego and those of its alters that show a considerable communication hiatus-transient relationships. Our results show there is a large range of relationship lifetimes for which communication volume displays no dominant trend, with longer lifetimes associated with larger volumes of communication. One interpretation that emerges from the lack of dominant trends is that relationship end cannot be inferred from a decay in calling. A considerable fraction of relationships begin with a period of more frequent contact before settling into their long-lasting pattern, a result particularly well supported by the UK and IT$_n$ cohorts made up of new alters. Finally, these effects are sufficiently robust that, over the various countries, ages, and life circumstances of our three cohorts, call volume at an early period of communication is found to contain a considerable amount of information about relationship lifetimes even across cohorts.

In terms of how transient relationships may fit into the picture of overall communication, we highlight the following aspects. First, in terms of the mechanics of pursuing relationship communication, the lack of systematic trends reported here for transient relationships is in line with our expectations of communication for long-term contacts^39^; in such relationships (say, with parents, relatives, or significant others) steady communication is needed. It may be that, *even if subjective evaluation of a relationship may be changing (e.g. decaying)*, it is more economical cognitively, or a better way to attain reciprocity, to have a temporal approach to communication with transient relationships that does not directly imitate the subjective evaluation. Second, as can be appreciated from Figs. 1 and 2, even relationships that only last 5 or 6 months have communication volumes that are substantial (roughly between 1 to 3 calls every 15 days), meaning that transient relationships do not typically constitute meaningless links. This does not, of course, mean that all relationships have such limited lifespans; a few of those with a slighlty larger call volume can last a lifetime, i.e. are non-transient. Saramäki et al.^15^ noted that, among 18-20 year-olds, turnover in friendships could be extremely high: only 40% of the alters retained their relative rank in terms of communication activity over an 18-month sample. More generally, a 10-15% of alters left or joined a network in any given year. The data for the US and Italian samples suggest that similarly high rates may be observed in older age cohorts in their later 20s and into their 30s. Longer term studies have also shown a high degree of turnover, with only 27% of close ties remaining after a decade in Canadian adults^6^. However, as our own results show, these transient alters take up a *substantial portion* of ego’s communication. Overall, this clearly illustrates the fact that contrary to what is often supposed relationship turnover is rather high in human relations, and the explanation of this effect is an important outstanding issue.

Another consideration emerging from our study concerns the complementary nature of objective communication information and subjective measures of relationships. Thus, although the use of Call Detail Records avoids some of the shortcomings that have been previously identified in self-reported patterns of communication^6,40–42^, including limited time resolution and poor recall effects, questionnaire or interview data are the only sources of subjective relationship measures (e.g. emotional closeness) and are therefore critical. In fact, the discrepancy between subjective relationship intensity’s decay over time and the absence of systematic decaying communication trends observed here indicates that *both* approaches are necessary to develop a full picture of an ego’s mechanisms in navigating social network creation, maintenance, and modification. To further clarify this, future studies should contemplate dimensions such as face-to-face contact, which has been previously associated with further longevity in relations^4^, and sampling of the reasons why people effectively cease to communicate with their alters. Indeed, understanding the interplay between objectively and subjectively measured relationship characteristics may be relevant to understand a variety of aspects of human communication, including how transient and long-term relationships are associated with well-being^2^.

It is reasonable to think that our definition of transient relationships will require further qualitative and quantitative studies because, as research into short- and long-term romantic relationships demonstrates^16^, there are likely to be a variety of reasons why transient relationships exist and why they end. In addition, the monotonic relation between volume of communication and lifetime we observe cannot be absolute, i.e. at some point, an increase in $\ell$ cannot lead to a further increase in *b* because this would mean that for large enough $\ell$, long transient relationships would in fact take up all available communication time. Thus, it would be important to learn at what point $\ell$ does not lead to further increases in *b* and, indeed, whether or not *b* stabilizes or maybe even starts to decrease with very stable (yet still possibly transient) relationships.

At a practical level, our results also have implications in designing research protocols, because they suggest that even relatively short time series of mobile data (approximately between 100 and 180 days, but above the $\ell _s$ limit) are sufficient to distinguish among alters who will go on to have different lifetimes in the network over a longer time period. As participant drop-out is a key issue in longitudinal studies^43,44^, this finding may enable researchers to design studies that optimize the balance between the length of the study and the likelihood of participant drop-out.

Whilst we found robust relationships between early call volume and lifetime in transient relationships in all three countries, there were some limitations to this study that may have impacted our research findings. First, the focus of this study was on understanding the temporal patterns of communication in transient relationships independent of individual characteristics. Thus, factors such as gender^45,46^, personality^47,48^, or whether a relationship is between friends or romantic partners^36,46^, may all affect these temporal patterns of communication. Therefore, future research could examine how ego and alter characteristics may modify the patterns we have identified. Second, given our initial motivation for testing whether gradual decay in subjective ratings also translated into objective gradual decay in communication volume, we focused our study on patterns of call volume. However, as has been shown in the context of related questions^24^, different characterizations of temporal signals may be informative. In the future, an expanded exploration of different temporal characterizations of communication in transient relationships may provide further valuable information about how such relationships evolve. Third, the lack of data that couples high temporal resolution subjective ratings with call patterns prevents us from understanding subjective ratings at a level of detail equivalent to that of calling data. Until such data are available, our understanding of the mismatch between objective and subjective measurement of transient relationship temporal behavior will remain unclear.

Another question pertains to patterns of communication as these increasingly shift from mobile calls and texts to messaging platforms and social media sites such as Whatsapp^34^, Twitter, Instagram^35,49,50^, and WeChat^51^. The diversity of these platforms makes collecting communication data more complex than relying solely on mobile data, but the development of applications that passively collect accurate data on mobile application use provides new opportunities for research in this area^52–54^. This variety of platforms and channels is not relevant to the present study due to the time frame when our data were collected (before the widespread use of smartphones in the respective countries). However, based on the fact that communication regularities seen in phone calls also appear in channels such as email^55^ and Facebook^56^, once the various channels of communication are aggregated, the overall signal may show a great deal of similarity with our present findings.

The connection between early call volume and lifetime of transient relationships may suggest support for a description of the effect of homophily in relationships called the “Seven Pillars of Friendship.” This description is made up of a set of seven cultural dimensions that define the individual and the cultural community they belong to^18^. These dimensions include: dialect, place of origin, career trajectory, hobbies/interests, moral/religious views, musical tastes, and sense of humour. Friendship quality has been shown to depend on the number of these friendship dimensions that an ego and a particular alter share^57^, reflecting the extent to which friendships are dominated by homophily – the tendency for ’birds of a feather to flock together’^19,57,58^. It has been suggested that, after first meeting, dyads initially devote time to checking out each others’ respective positions on the seven pillars, and then adjust their rate of contact to that appropriate for the quality of relationship defined by the number of pillars they share^31,58^. Evaluating our results against this proposal, a number of areas of consistency emerge. First, note that call volume measured in the early part of a relationship (e.g. the second month) has predictive power about a relationship’s lifetime (Figs. 4 and 5). If the lifetime of a relationship was merely a consequence of a continuous evaluation in which, at any point, a relationship could be dissolved, call volume at the early part of a relationship would provide no information about lifetime (for example, Figs. 4 and 5 would not show differences due to early call volumes). A second consideration that may signal consistency between our results and the Seven Pillars of Friendship is the fact that many cohorts of different lifetime $\ell$ do exhibit a fast very early period of elevated volume of communication followed by a rapid decay, as visible from Fig. 1 and Fig. S8, near $a\approx 0$. Further study of this possible connection is warranted.

In summary, communication volume of egos to groups of similar transient alters is stable, with no signs of a dominant gradual decay of such call volume over lifetime. The volume of calls is associated with greater longevity of a transient relationship. These findings are consistent across three countries and for different demographic groups. Similar to a few other studies, we observe that the volume of communication egos invest in transient alters is far from negligible, suggesting that such relationships are essential. In a broader context, our results uncover a new striking regularity in ego networks that reinforces related findings^15,59–61^ of regularity and steadiness within the dynamics of communication.

## Methods

### Data

All the analyses are based on three mobile phone data sets: (i) the UK data comes from an 18-month ($T_{\textrm{UK}} = 546$) study of 30 students in their final year of secondary school, who were followed as they made the transition from school to university^36^; (ii) Friends and Family data set collected phone calls of 130 people from a residential community centered around a university in the US^62^ over a period of $\approx 17$ months ($T_{\textrm{US}} = 505$); and finally (iii) the Italy data set, containing phone calls collected from 142 parents with young children aged 0 through 10 years^38^ over a period of around 22 months ($T_{\textrm{IT}} = 699$). For each data set $\mathscr {E}=\{\textrm{UK, US, IT}\}$, we limit the ego-alter pairs used to those with a maximum duration of communication $\mathscr {L}_{\mathscr {E}}$ based on the point at which, due to study design and duration of each of the national studies, the percentage of egos with active relationships begins to decay significantly (see Supplementary Information, Sect. S1.2). The UK data set is further filtered (as explained in “Results” section) to ego-alter pairs that appear only after 6 months of the study, when participants begin university study. We also exclude relationships with less than 3 calls since such relations are uninformative. Further filtering is applied to determine transient relationships (see “Transient alter selection” section). Finally, the IT${}_n$ cohort is constructed by further filtering relationships to those in the Italian data that do not commence until after a minimum number of days since the entry of the participant. These filters define our four cohorts UK, US, IT, and IT${}_n$. All data sets were collected before smartphones became common and thus capture the bulk of people’s non-face-to-face communication.

### Transient alter selection

Each communication event (outgoing phone call) between ego *i* and alter *x* occurs on a particular day $a_{ix}$ after their first observed communication, where the first day corresponds to $a_{ix}=0$. From the perspective of when each cohort $\mathscr {E}$ begins, the first observed contact between *i* and *x* occurs on day $t^{(1)}_{ix}$ which is a number between 0 and $T_{\mathscr {E}} - 1$. If there are $n_{ix}$ total observed calls between *i* and *x*, the last call occurs on day $t^{(n_{ix})}_{ix}$ of the study, which corresponds to $\ell _{ix}=t^{(n_{ix})}_{ix}-t^{(1)}_{ix}$. In our study, we exclude any alter *x* such that $T_{\mathscr {E}} -t^{(n_{ix})}<\Delta t_w$ where $\Delta t_w$ is an excluded window that provides confidence that a relationship has indeed stopped communicating for a significant amount of time. A full explanation of the construction of cohorts is provided in the Supplementary Information, Sect. S1.4

### $\bar{f}_{i}(a, \ell )$ and $\bar{f}(a, \ell )$ definitions

The call volume $f_{ix}(a_{ix},\ell _{ix})$ between *i* and *x* captures the evolution of relationship *ix* over time, but it is a considerably noisy signal, generally with few samples for given values of $a=a_{ix}$ and $\ell =\ell _{ix}$. To address the possibility that egos have a systematic trend over time in communicating with their alters, we average over alters of *i* in $\mathscr {A}_i(\ell ,\Delta \ell )\subset \mathscr {A}_i$, the set of alters *x* such that $\ell \le \ell _{ix}<\ell +\Delta \ell$. The bin size in the main text has been chosen as $\Delta \ell =50$ days, but other values are shown in the Supplementary Information, Sect. S3.3. From these definitions, as well as a window of *a* such that $a\le a_{ix}<a+\Delta a$ (with $\Delta a=15$), we introduce $\bar{f}_i(a,\ell )=\sum _{x\in \mathscr {A}_i(\ell ,\Delta \ell )}f_{i,x}(a_{i,x},\ell _{i,x})/\vert \mathscr {A}_i(\ell ,\Delta \ell )\vert$. We also introduce $\bar{f}(a,\ell )=\sum _{i}\bar{f}_i(a,\ell )/\sum _{i}\theta (\vert \mathscr {A}_i(\ell ,\Delta \ell )\vert )$, where $\theta (\cdot )$ corresponds to the step function ($\theta (x)=1$ if $x>1$, and 0 otherwise), and $\vert \vert$ produces the cardinality of a set. Note that any trend consistently present in $f_{ix}(a_{ix},\ell _{ix})$ would be inherited by both $\bar{f}(a,\ell )$ and $\bar{f}_i(a,\ell )$.

### Kolmogorov-Smirnov test for $\bar{f}_{i}(a, \ell )$

We study the level of steadiness of $\bar{f}_i(a,\ell )$ as a function of *a* ego by ego, taking for each time series $\bar{f}_i(a,\ell )$ two parts of equal duration in *a* that exclude the first ($a=0$) and last ($a=\lfloor \ell /\Delta a \rfloor \Delta a$) points of the time series. These two points are excluded for specific reasons. The first point is affected by initial tendencies to have communication that has not stabilized, as can be seen in Fig. S8. The last point is excluded because, unless $\ell$ is a perfect multiple of $\Delta a$, the call volume captured by the last time point of the series is likely to have less call volume simply because it is not fully used (there is a period between $\ell$ and $\lfloor \ell /\Delta a\rfloor \Delta a$ with no activity). After excluding these two points, the two resulting ranges of elapsed duration ($\Delta a\le a< \lfloor (1/2)\left( \lfloor \ell /\Delta a\rfloor -1\right) \rfloor \Delta a$ and $\lfloor (1/2)\left( \lfloor \ell /\Delta a\rfloor -1\right) \rfloor \Delta a\le a\le \lfloor \ell /\Delta a \rfloor \Delta a-\Delta a$) generate for each ego two samples of $\bar{f}_i(a,\ell )$ at points in *a* within each of the periods, and we perform a Kolmogorov-Smirnov test to determine if the values of the two samples come from the same distribution. The result of the Kolmogorov-Smirnov test for each ego is a *p*-value that, the closer it is to 1, the more likely it is that the series $\bar{f}_i(a,\ell )$ is steady. Let us label the *p*-value obtained for each ego as $p_i$. We conduct these tests for egos with medium and long lifetimes. Figure 3A shows box plots of the $\{p_i\}_{i\in \mathscr {E}}$ obtained from the tests for all cohorts $\mathscr {E}$.

### $b(\ell )$, $b_i(\ell )$, and $\ell _s$ computation

The determination of $b(\ell )$, $b_i(\ell )$, and $\ell _s$ is made by identifying a *stable region average* of $b(\ell )$ (a second method is also used in the Supplementary Information, Sect. S4 with similar results as here). In order to obtain this average, we find the longest range of values of *a*, pivoted around the center of the range, where $\bar{f}(a,\ell )$ or $\bar{f}_i(a,\ell )$ is steady (flat) in *a*. In this description, we label both $\bar{f}(a,\ell )$ and $\bar{f}_i(a,\ell )$ as *u*(*a*), where $\ell$ is not written to avoid complicating the notation but it is implied in that $a\le \ell$. The criterion to determine if *u*(*a*) is close to flat is based on whether its average slope oscillates around 0. This flatness is tested iteratively between two values of *a*, $a_m$ and $a_M$, which must be found by the method. The algorithm starts with $a_m=0$ and $a_M=\lfloor \ell /\Delta a\rfloor \Delta a$, and alternatively and iteratively increases $a_m$ while leaving $a_M$ fixed and, in the next step, decreases $a_M$ while leaving $a_m$ fixed, and so on. The changes in both $a_m$ and $a_M$ are done in increments of $\Delta a$. The algorithm stops when the average slope of *u*(*a*) starts to oscillate around 0, or if no stable region is found. The method takes advantage of the fact that typically when *u*(*a*) does reach a stable regime, only the regions near $a=0$ and $a=\ell$ substantially deviate from being flat and are each only a few units of $\Delta a$ in the range of *a*. The concrete application of the method is as follows. Let the values of *a* for which we calculate *u*(*a*) be given by $a=\alpha \Delta a$ with $\alpha$ an integer between 0 and $\lfloor \ell /\Delta a\rfloor$. Using integer *q*, we calculate the slope

2 $$\begin{aligned} \mathrm{average\;slope}(q)=\frac{u\left( \lfloor \frac{\ell }{\Delta a}\rfloor \Delta a -\left\lfloor \frac{q+1}{2}\right\rfloor \Delta a\right) -u\left( \left\lfloor \frac{q}{2}\right\rfloor \Delta a\right) }{\left( \lfloor \frac{\ell }{\Delta a}\rfloor -q\right) \Delta a } \end{aligned}$$

for each value of *q*, starting at 0, and increasing in increments of 1 until the sign of the average slope (Eq. 2) first alternates twice in consecutive values of *q*, or until $q=\lfloor \ell /\Delta a\rfloor$ if the alternation condition is never met. Note that $\lfloor q/2\rfloor \Delta a$ and $\left[ \lfloor \ell / \Delta a\rfloor -\lfloor (q+1) / 2\rfloor \right] \Delta a$ correspond to two values of *a* roughly equidistant to the center (one to the left and one to the right) of the range of *a* for *u*(*a*). These two values are labelled $a_m(q)=\lfloor q/2\rfloor \Delta a$ and $a_M(q)=\left[ \lfloor \ell / \Delta a\rfloor -\lfloor (q+1) / 2\rfloor \right] \Delta a$ as indicated before. The increase in *q* one unit at a time increases $a_m$ to $a_m+\Delta a$ in one step while leaving $a_M$ unchanged, and in the next step decreases $a_M$ to $a_M-\Delta a$ while leaving $a_m$ unchanged. This process truncates the two ends of the range of values of *u*(*a*) over which the average slope is being calculated. If alternation of the sign of Eq. (2) occurs for two consecutive increases of *q*, i.e. when *q* changes from value $q_x$ to $q_x+1$ and from $q_x+1$ to $q_x+2$, we take $q_x$ as the beginning of the approximately 0-average slope of *u*(*a*). If the average slope sign alternation condition is never met, or if the average slope is always identical to 0, the algorithm stops when the range of *a* cannot be truncated any further, which is when $q=\lfloor \ell /\Delta a\rfloor$, and in this case, we make $q_x=2$ which means that we revert to looking at all but the two endpoints of *u*(*a*) (although the algorithm is deemed to have failed to converge and we use its results differently). Using the resulting $q_x$ (converging or non-converging), we measure $\bar{u}(a)$, the average of *u*(*a*), using all $u(\alpha \Delta a)$ with $\left\lfloor q_x / 2\right\rfloor \le \alpha \le \lfloor \ell / \Delta a\rfloor -\left\lfloor (q_x+1) / 2\right\rfloor$. When *u*(*a*) corresponds to $\bar{f}(a,\ell )$, then $b(\ell )=\bar{u}$; when *u*(*a*) corresponds to $\bar{f}_i(a,\ell )$, then $b_i(\ell )=\bar{u}$.

The method described above also yields the minimum lifetime $\ell _s$ at which stable regions begin to emerge. As noted above, $q_x=2$ when the method does not converge, otherwise, the method converges and therefore, at $a=\lfloor q_x/2\rfloor \Delta a$ a flat region of *u*(*a*) begins. Noting that this *a* is equivalent to the shortest possible value of lifetime, we equate $\ell _s$ with $\lfloor q_x/2\rfloor \Delta a$ and take *u*(*a*) to be $\bar{f}_i(a,\ell )$. This produces a sample of $\ell _s$, one for each ego, and provides a statistical picture for the smallest lifetimes that exhibit a steady regime. We study $\ell _s$in the Supplementary Information, Sect. S5.4.

### $P(a\mid a_o,a_f,\gamma )$ computation

The probability $P(a\mid a_o,a_f,\gamma )$ of a relationship continuing to be active to at least elapsed duration *a*, with a number of calls *g* that falls in bin $\gamma$ during the window $a_o\le a\le a_f$, is calculated over a set of transient ego-alter relationships with $0 \le \ell \le \mathscr {L}_{\textrm{US}}$ (the smallest value of largest $\ell$ possible among the cohorts in the figure). Concretely, if the total number of alters that receive *g* calls (falling in bin $\gamma$) in the window between $a_o$ and $a_f$ is $N(a_o,a_f,\gamma )$ and only $N(a;a_o,a_f,\gamma )$ out of those are still communicating at some $a>a_o$, then $P(a\mid a_o,a_f,\gamma )= N(a;a_o,a_f,\gamma )/N(a_o,a_f,\gamma )$. Bins for $g$ are exponentially spaced, corresponding to the ranges $[3^0,3^1);\dots ;[3^4,3^5]$ and $\gamma$ is the exponent of the minimum power of 3 that identifies each bin.

### Mutual information

The measurement of mutual information between the random variables $\ell$ and *g* is performed for all the combined cohorts together and also for individual cohorts. Mutual information $I(\textbf{X},\textbf{Y})$ between two random variables $\textbf{X}$ and $\textbf{Y}$ is defined as the amount of information one of the random variables contains about the other. Specifically, for discrete random variables,

3 $$\begin{aligned} I(\textbf{X},\textbf{Y})=\sum _{x\in \textbf{X},y\in \textbf{Y}}\textrm{Pr}(\textbf{X}=x,\textbf{Y}=y)\log _2\left[ \frac{\textrm{Pr}(\textbf{X}=x,\textbf{Y}=y)}{\textrm{Pr}(\textbf{X}=x)\textrm{Pr}(\textbf{Y}=y)}\right] , \end{aligned}$$

where $\textrm{Pr}(\textbf{X}=x,\textbf{Y}=y)$ is the joint probability to draw *x* and *y* simultaneously, $\textrm{Pr}(\textbf{X}=x)$ the marginal probability to draw *x*, and $\textrm{Pr}(\textbf{Y}=y)$ the marginal probability to draw *y*. $I(\textbf{X}, \textbf{Y})$ is measured in *bits*, which we can normalize to a *symmetric uncertainty* $U(\textbf{X}, \textbf{Y})$,

4 $$\begin{aligned} U(\textbf{X}, \textbf{Y}) = \frac{2I(\textbf{X}, \textbf{Y})}{H(\textbf{X}) + H(\textbf{Y})}, \end{aligned}$$

where $H(\textbf{X})$ and $H(\textbf{Y})$ are the entropies of $\textbf{X}$ and $\textbf{Y}$, respectively, defined as $H(\textbf{X})=-\sum _{x\in \textbf{X}}\textrm{Pr}(\textbf{X}=x)\log _2 \textrm{Pr}(\textbf{X}=x)$ (and similarly for $H(\textbf{Y})$). The advantage of using $U(\textbf{X}, \textbf{Y})$ is mostly its interpretation. When the two variables are independent, $U(\textbf{X}, \textbf{Y}) = 0$, and when there is complete information about one variable from the other, $U(\textbf{X}, \textbf{Y}) = 1$.

### Computational and statistical tools used

In this article, most of the statistical functions employed have been programmed from scratch, using Python 3.10. For some particular uses, the following Python packages were used: for data cleaning (applying all filters described above to identify transient relationships), pandas 1.5.1; for some mathematical functions required to create histograms, numpy 1.23; for KS tests and OLS estimations, statsmodels 0.13; for mutual information tests, scikit-learn 1.1.

## Supplementary Information

Supplementary Information.

## Data Availability

The US data can be accessed through the Reality Commons database (MIT) as the Friends and Family data (http://realitycommons.media.mit.edu/friendsdataset.html). The UK data relevant to this study has been made available previously in the publication Saramäki et al. (2014) “Persistence of social signatures in human communication”, PNAS 111 (3) 942-947. The Mobile Territorial lab data used in this study are not freely available on an open repository for privacy reasons. However, they are available upon request by contacting the authors at lepri@fbk.eu. The data will be made available in a timely manner and in compliance with any ethical or legal requirements.
