# Supplementary material for: The stability of transient relationships
Source: Sci Rep. 2023 Apr 14;13:6120. doi: 10.1038/s41598-023-32206-2 (PMC10104882; doi:10.1038/s41598-023-32206-2)
Supplement: Supplementary file 1 — Supplementary Information. [file 41598_2023_32206_MOESM1_ESM.pdf]

Supplementary Information for The Stability of Transient Relationships

Valentín Vergara Hidd[1,*,+], Eduardo López[1,+], Simone Centellegher[2], Sam G. B. Roberts[3], Bruno Lepri[2], and Robin I. M. Dunbar[4]

[1]George Mason University, Computational and Data Sciences Department, Fairfax, 22030, US
[2]Fondazione Bruno Kessler, Mobile and Social Computing Lab, Trento, I-38123, Italy
[3]Liverpool John Moores University, School of Psychology, Liverpool, L3 3AF, UK
[4]University of Oxford, Department of Experimental Psychology, Oxford, OX2 66G, UK
[*]Corresponding Author: vvergara@gmu.edu
[+]these authors contributed equally to this work

As a general note, in this document the term relationship between ego-alter pairs refers to *transient relationships*. In many instances, we simply write relationship for brevity, but this should always be understood to mean transient relationships. The *only exception* to this rule is encountered in Sec. S1.2. and its only figure (Fig. S1).

S1 Construction of cohorts for each study

A succinct explanation of the data is provided in the main text (Sec. Methods). These data sets have been fully described in previous articles, and the corresponding citations can be found below in the respective subsections.

Here we expand on some details of the studies that generated these data, namely the timing of entry of participants into the study and the life circumstances of the participants in each study. These details affect the way in which we choose the transient relationships we analyze in this article. After these descriptions, we elaborate on the filters we apply to arrive at the final cohorts in this study.

S1.1 Detailed information about each National study

S1.1.1 UK study

The UK dataset was collected between 2007 and 2008, with all participants (egos) starting the experiment simultaneously. The timing of data collection was chosen to start observing participants during the last few months of secondary school and then continue to observe them for a time period that would capture an entire first year of university study. All participants were recruited from the same cohort in one school. The transition from secondary school to university occurs around six months after the start of data collection. With this design, participants begin the study while interacting with generally well-established network members (alters), and after six months, participants start to engage with a host of contacts that can be taken to be newly met alters (for details see[1]). Of the total cohort of 30, only 2 participants did not transition to university but their networks were still deeply disrupted due to the loss of alters and new personal circumstances.

S1.1.2 US study

The US dataset was collected between 2010 and 2011, with a pilot phase lasting 6 months, and then a second phase of 12 months in which an additional larger pool of participants is recruited at the beginning of this phase. This means that the egos did not all start simultaneously (for details see[2]). Effectively, this produced a sample of approximately 17 months due to some overlap between the pilot and remaining phases of the study.

The circumstances of the participants (egos) in the US data set are generally steady in time, i.e. the participants were not intentionally recruited to capture a particularly large change in their circumstances. Furthermore, egos do not generally enter synchronously in the phases of the study.

S1.1.3 Italian study

The Mobile Territorial Lab experiment recruited participants in two groups, one beginning their participation in early 2013 and the second in early 2014[3]. As with the US dataset, egos were not selected to be in a particularly dynamic stage of their lives where large changes to their circumstances could be foreseen. As with the US data set, egos in this study do not begin synchronously.

S1.2 Bounds on lifetimes by cohort

The numbers of ego-alter relationships in all data sets decrease as a function of observed lifetimes. These decreases begin at a steady rate for all data sets, but as the lifetimes start to reach values that resemble the duration of the study, and in most cases considerably less time, the number of ego-alter pairs drop off at increased rates.

Fig. S1 shows the situation for all three national studies used. In each study, if an ego has at least one relationship that is still active at elapsed duration a (horizontal axis), we count that ego into the proportion of active egos in the study; otherwise, the ego does not count. The proportion is displayed on the vertical axis. To choose the longest lifetimes $\mathscr{L}_{\mathscr{E}}$ used in each of the studies, we locate the value of a at which the proportion of egos with at least one relationship active at a starts to decay at an increased rate. This filtering prevents the possibility of attempting to build statistical inferences about lifetimes (particularly those with values close to $\mathscr{L}_{\mathscr{E}}$), on the basis of one or two ego-alter pairs per ego. The specific $\mathscr{L}_{\mathscr{E}}$ values are indicated in the plot by vertical dashed lines, and correspond to $\mathscr{L}_{\mathscr{E}}$ of the respective country $\mathscr{E}$. The values in days, reported in the plot, are $\mathscr{L}_{\mathrm{UK}} = 270$ days, $\mathscr{L}_{\mathrm{US}} = 220$ days, and $\mathscr{L}_{\mathrm{IT}} = 365$ days. For the US, we choose to take a slightly larger $\mathscr{L}_{\mathrm{US}}$ than strictly supported by the plot (specifically 20 days) because otherwise long lifetimes for the US become comparable to medium lifetimes of other cohorts on the basis of the rules we have chosen for selecting lifetime groups (see main text, discussion of Fig. 1). However, this adjustment is quite minor, and robustness checks presented below in Sec. S3 show that any error that this may lead to is not appreciable.

With all the values of $\mathscr{L}_{\mathscr{E}}$ on hand, the ego-alter pairs we study are those that satisfy

$$\ell_{ix} \leq \mathscr{L}_{\mathscr{E}}, i \in \mathscr{E}. \tag{1}$$

S1.3 Calculation of initial relationship times for asynchronous ego entry

The US and Italian studies add participants in an asynchronous way, i.e. not all participants become active at the same time. This becomes relevant for some measurements presented in the main text and in this supplementary document.

To deal with this effect, for each ego i in both the Italian and US data sets, we define an entry time ε_i, equal to the day t of the study that i belongs to when this ego is seen to make the first contact with any of its alters $\mathscr{A}_i$ (defined in the Methods, main text). Then, for ego-alter pair ix, we generate a relative start day $\tau_{o,ix} = t_{ix}^{(1)} - \varepsilon_i$, where $t_{ix}^{(1)}$ is the day of first contact between i and x measured from the first day of the study to which i belongs. Thus, $\tau_{o,ix}$ measures the number of days since ego i entered his/her study before i and x were observed to begin contact.

We use this information in two different ways. First, we use it in determining how many transient relationships in each national study begin at or after a certain number of days from the entry of an ego into the study (see Sec. S2). The second way we use this information is the construction of the IT_n cohort, described next.

S1.4 Construction of cohorts

We develop four cohorts. Two of the cohorts (UK and IT_n) are meant to characterize transient relationships where there is a high chance the initial contact is observed within the data. Two other cohorts (IT and US) are not adjusted to specifically try to capture the initial contact of ego-alter pairs but, as we see in Sec. S2, this occurs for most contacts due to chance.

All the cohorts explained next satisfy the following: i) each ego-alter pair ix has at least 3 contacts, so as to avoid studying meaningless relationships, ii) no lifetimes ℓ_{ix} are larger than the $\mathscr{L}_{\mathscr{E}}$, where $i \in \mathscr{E}$, and iii) ego-alter pairs comply with the transient relationship filter Δt_w, explained in the main text.

The Italian (IT) and US cohorts are fully defined by the three filters just mentioned. The other two cohorts satisfy additional conditions.

S1.4.1 Construction of UK cohort

Beyond the conditions stated above, the UK cohort is generated by only using ego-alter pairs that become active after 6 months or more from the start of the study. All egos have activity and therefore the cohort has all egos that enter the study from the beginning. Alters seen entering ego networks at that point are believed to be almost all new.

S1.4.2 Construction of IT_n cohort

This sub-cohort of the Italian cohort, in addition to conditions i, ii, and iii, includes a filter such that an ego-alter pair ix is used only if $\tau_{o,ix} \geq \Delta t_s$, where Δt_s is an exclusion window at the start of a participant's time in the study. This condition means that if pair ix is active before the ego has been in the study at least Δt_s days, the relationship is ignored. This filter is a way to reduce the number of ego-alter pairs analyzed for IT_n that may have been active before the actual start of the study. As we show in Sec. S2, a large portion of transient relations actually begin after the start of the study and therefore, the filter introduced by using Δt_s further lessens the likelihood of using an ego-alter pair that was in communication before the start of the study.

The result of the application of all the filters stated above is cohorts where the sample size is indicated in Table 2 of the main text.

S2 Starting and ending times of relationships inside each study

As explained in the main text, even though in the US and Italian studies, some ego-alter pairs may have been active before the start of the study, the majority of transient relationships begin well after an ego enters his/her respective study (see next). This goes a long way in explaining why the various analyses we undertake in this study work similarly well when using UK and IT_n or US and IT.

To provide evidence for this interpretation, we present the cumulative distribution for τ_o (that is, the random variable associated with the individual $\tau_{o,ix}$ for concrete ix pairs), separated by cohorts (Fig. S2A). Each curve shown provides, per cohort, the percentage of ego-alter pairs that are first seen to be active on or before day τ_o. Clearly, although many relationships are first observed for small values of τ_o, they are by no means the majority. For instance, in most studies, 40% of relationships require that τ_o reaches a value of ≈ 50 days. The US is the only exception, starting at $\approx 40\%$ when τ_o is still quite small. By contrast, IT_n requires well over 100 days to reach 40% of transient relationships.

A similar analysis can be carried out with regards to the final contact between ego i and alter x, which takes place $T_{\mathscr{E}} - t_{ix}^{(n_{ix})}$ days before the end of each study. This is relevant when assessing how close to the filter Δt_w each pair ix gets. As Fig. S2B shows, this filter is not commonly reached. Thus, for example, in most cohorts only about 40% of ego-alter pairs remain in touch when there still over 100 days before the end of the study.

These two analyses provide support for the consistency seen in the results coming from cohorts UK, IT_n, IT, and US because, although for the first two a,ℓ more strictly measure the actual elapsed duration and lifetime of transient relationships than the latter two, in practice many transient relationships are well contained inside the time boundaries of the studies, effectively making all four cohorts similar.

S3 Robustness check for $\bar{f}(a,\ell)$

This section is concerned with robustness checks for the results and interpretation of $\bar{f}(a,\ell)$, presented in Fig. 1 of the main text. In summary, the results in the next subsections support the robustness of our conclusions about $\bar{f}(a,\ell)$, i.e. that for lifetimes of enough duration (see Sec. S5 regarding this point) $\bar{f}(a,\ell)$ is indeed steady over a until it ceases (***the steadiness feature***), and that $\bar{f}(a,\ell)$ generally increases as a function of ℓ (***the monotonicity feature***). After showing the standard error for each point of $\bar{f}(a,\ell)$, each subsection focuses on testing the effect of each particular measurement parameter ($\Delta t_w, \Delta\ell, \Delta a$) on $\bar{f}(a,\ell)$, with an additional subsection that tests $\bar{f}(a,\ell)$ against Δt_s in IT_n.

S3.1 Standard error of each value of $\bar{f}(a,\ell)$

The results presented in Fig. 1 in the main text consider the stable volume of communication for the aggregated alters and egos of a given lifetime range. In order to show that variation exists at the individual level, we support our results with those from Fig. 3B in the main text. Additionally, as an alternative visualization for the variation among individual egos, we show one standard error for each point of $\bar{f}(a,\ell)$ in Fig. S3 as shaded regions in a color corresponding to their lifetime group.

For all cohorts, as lifetime increases, so does the standard error. This is due to the fact that the number of transient alters decreases with ℓ, as seen in Fig. S1.

S3.2 Decay in $\bar{f}(a,\ell)$ for a between ℓ and $\ell+\Delta\ell$

Our method for measuring $\bar{f}(a,\ell)$, although effective in terms of generating a reliable estimate of the per ego per alter calling volume of each ego to their alters, also has the unintended consequence of frequently generating a fast decaying tail for $\bar{f}(a,\ell)$ for $\ell \le a \le \ell+\Delta\ell$ visible in medium and long lifetimes (Fig. 1 of the main text as well as figures of the robustness checks of the current section). Here, we explain the origin of this effect, which is *not* a behavioral feature of egos, but rather a statistical nuisance effect.

As defined in the main text, $\bar{f}(a,\ell)$ is given by

$$\bar{f}(a,\ell) = \frac{\sum_i \bar{f}_i(a,\ell)}{\sum_i \theta(|\mathscr{A}_i(\ell,\Delta\ell)|)}, \tag{2}$$

where $\theta(\cdot)$ corresponds to the step function ($\theta(x)=1$ if $x>1$, and 0 otherwise), and $||$ produces the cardinality of a set. In the range of a starting with ℓ and ending at $\ell+\Delta\ell$, there is a progressive reduction of the numerator of Eq. 2 that occurs because not all individual egos have alters until $a=\ell+\Delta\ell$. Instead, any given ego typically has activity until a value of a somewhere in the middle of the range between ℓ and $\ell+\Delta\ell$. Let us assume that the ego in question is i. If the last active alter with lifetime between ℓ and $\ell+\Delta\ell$ in ego i's network stops activity at $a_i^{(\mathrm{end})}$, then the time series $\bar{f}_i(a,\ell)=0$ for $a>a_i^{(\mathrm{end})}$. However, in Eq. 2, the denominator is unchanging, which means that between $a_i^{(\mathrm{end})}$ and $\ell+\Delta\ell$, $\bar{f}(a,\ell)$ is calculated with the same denominator but a diminished numerator with no contributions from ego i. Crucially, the distribution of end-times for each of the time series $\bar{f}_i(a,\ell)$ occurs all throughout the range between ℓ and $\ell+\Delta\ell$.

In Fig. S4, we show the number of egos still active in the range between ℓ and $\ell + \Delta\ell$ respective to each of the cohorts shown in Fig. 1 of the main text. As it is clear from these plots, the number of active alters decays rapidly from a value of $|\mathscr{A}_i(\ell, \Delta\ell)|$ to 0 causing $\bar{f}(a, \ell)$ to also decay within this temporal range. This effect leads to the generation of the fast drops seen in most of the curves in Fig. 1. However, we should note that the decay can be partially attenuated if, by random chance, a group of egos in some lifetime group ℓ to $\ell + \Delta\ell$ remains active until closer to $\ell + \Delta\ell$ and/or the total call volume among those egos near the end of the time series fluctuates upwards (see e.g. medium lifetime for Italy in Fig. 1).

S3.3 Robustness in Δt_w

In our study, we exclude any alter x such that $T_{\mathscr{E}} - t_{ix}^{(n_{ix})} \leq \Delta t_w$ which means that we study ego-alter pairs that stop communicating at some point in the study and remain without communication until the end of the study and for a minimum of at least Δt_w days. This is effectively our transient relationship operational criterion.

The main text presents results with $\Delta t_w = 60$ (fourth row in Fig. S5). Here, we test robustness by also checking $\Delta t_w = 10, 30, 50, 90$. As Fig. S5 shows, using different values of Δt_w does not affect either the steadiness nor the monotonicity features.

S3.4 Robustness in $\Delta\ell$

The value of $\Delta\ell$ of each lifetime group in Fig. 1 of the main text has been chosen as $\Delta\ell = 50$ days (circles in Fig. S6). Testing $\Delta\ell = 10$, $\Delta\ell = 30$, $\Delta\ell = 70$, and $\Delta\ell = 90$ leads to consistent results for medium and long lifetimes in terms of steadiness and monotonicity of $\bar{f}(a, \ell)$. An interesting observation also emerges for short lifetimes where ℓ is below the threshold value ℓ_s (see Sec. S5.4) for steady behavior: as $\Delta\ell$ increases, $\bar{f}(a, \ell)$ begins to change from a decaying behavior to one that develops a steadier range over values of a, signalling a trend towards steadiness.

S3.5 Robustness in Δa. Estimation of a_s

In the main text, Fig. 1 uses $\Delta a = 15$. Fig. S7 shows different values of Δa (Δa = 5, 10, 15, 30, 45). Small Δa leads to $\bar{f}(a, \ell)$ with more fluctuations, while large Δa exhibits very steady features. In all cases, the qualitative features of $\bar{f}(a, \ell)$ are preserved, in terms of the steadiness of communication to alters with medium and long lifetimes.

In addition to the Δa above, we also apply $\Delta a = 1$ to estimate the value a_s. However, given that this quantity appears across values of ℓ, we use $\bar{f}(a, \ell \geq \ell_s)$. This has the additional advantage of improving our sample size. As Fig. S8 shows, the first set of points on the plot, $a = 0, 1$, and 2, all show a steady decreasing trend before the curve begins to stabilize. Therefore, we believe that $a_s = 2$ constitutes a lower bound for the applicability of Eq. 1 in the main text.

S3.6 Robustness in Δt_s for IT_n

To construct IT_n in the main text, we use $\Delta t_s = 50$. Although most of our subsequent analysis in the main text as well as in this supplementary document (Sec. S2) supports the idea that $\bar{f}(a, \ell)$ is robust even if the start of a transient relationship is not captured, we nevertheless test for this robustness. In Fig. S9, we present $\bar{f}(a, \ell)$ calculated for $\Delta t_s = 30, 40, 50$ and find consistent results, supporting the steadiness and monotonicity features. Further filtering of Δt_s reduces the sample considerably and thus becomes unreliable for values $\Delta t_s \geq 50$.

S3.7 Volume and duration of calls

To study the temporal signal of communication, one can study numbers of calls or call durations. However, these two choices are known to be correlated for the UK data we use here[4]. In order to provide a full picture of this correlation focused on transient relationships, we measure the Person correlation of total numbers of calls and total time spent communicating between each ego-alter pair in all the our cohorts, and obtain the following results: the combined cohort shows a correlation of $r = 0.6544$, while the cohorts coefficient are: $r_{\mathrm{UK}} = 0.5739$; $r_{\mathrm{IT_n}} = 0.8238$; $r_{\mathrm{IT}} = 0.8769$; and $r_{\mathrm{US}} = 0.2332$. Fig. S10 shows scatter plots for each of the cohorts, in which each point corresponds to the total number of calls and total time ego spent talking to one of its transient alters. These results support our choice of only focusing on number of calls as a useful metric, as call duration would produce redundant analysis.

S4 Determination of $b(\ell)$, and $b_i(\ell)$

The height of the plateaus of each $\bar{f}(a, \ell)$ associated with a set of alters of a given range of lifetimes ℓ to $\ell + \Delta\ell$ in a cohort $\mathscr{E}$ is measured by $b(\ell)$. Similarly, the set of $b_i(\ell)$ captures the heights of the plateaus of individual egos' $\bar{f}_i(a, \ell)$. In this section, we discuss an alternative method to obtain $b(\ell)$ and $b_i(\ell)$ to the *stable region average* presented in the main text, Methods section.

S4.1 Mann-Kendall method to identify $b(\ell)$

As an alternative to the *Stable Region Average* method presented in the main text, here we give an alternative calculation of $b(\ell)$, using the Mann-Kendall test[5,6], explained further in[7], to detect trends in the data. We use the `Python` implementation provided by[8]. The basic intuition of the test can be understood as a simplification first proposed by Mann[6] of the Kendall rank-correlation test[5]. In particular, for a signal such as $\bar{f}(a,\ell)$ or $\bar{f}_i(a,\ell)$ (which for generality we denote as $u(a)$), one defines a test statistic $S = \sum_a \text{sign}(u(a+\Delta a) - u(a))$, where $\text{sign}(u(a+\Delta a) - u(a)) = 1$ if $u(a+\Delta a) > u(a)$, $= 0$ if $u(a+\Delta a) = u(a)$, and $= -1$ if $u(a+\Delta a) < u(a)$. The null hypothesis is for there to be no trend, in which case S is a normally distributed random variable with mean 0. Normality of the input random variable is not a requirement of this non-parametric test. To apply the test, we truncate the range of a from the left and from the right systematically as in the stable region test (see subsection "$b(\ell)$, $b_i(\ell)$, and ℓ_s computation" in the main text), stopping when no trend is detected (slope tends to 0), i.e. when the null hypothesis of the test can no longer be rejected at a significance level of 0.05. This procedure identifies the steady regions of $u(a)$.

S4.2 Results from application of the Mann-Kendall method

In this section, we show results for $b(\ell)$ done across the values of ℓ with the Mann-Kendall method described above. This result can be seen in Fig. S11. From the plot, we see that $b(\ell)$ increases with ℓ almost universally, with the exception of minuscule fluctuations early in IT and IT_n, and again for the longest ℓ for IT_n. This last deviating point occurs because the introduction of Δt_s effectively eliminates a great deal of the ego-alter samples that are available for equivalent lifetimes of IT, thus reducing statistical sampling. Overall, the trends are clear and highly consistent across cohorts (see Fig. 2 in the main text).

The fact that $b(\ell)$ is increasing with ℓ also supports the claim made in the main text that the selection of the medium and long lifetimes used in Figs. 1 and 3 is mostly arbitrary and for the purposes of illustrating the behavior of $\bar{f}(a,\ell)$ for concrete values of ℓ. However, these choices of ℓ are not restrictive and in fact one can work with values of ℓ from ℓ_s and up.

One last observation is that, while the trends of $b(\ell)$ are increasing, there are differences among the cohorts, with the US and UK showing a more rapid growth than the Italian cohorts, which start roughly steady and then begin their marked increase for larger values of ℓ. This may have implications in terms of how effectively one can distinguish medium lifetimes in Italian ego-alter pairs in comparison to the other cohorts on the basis of early phone call activity. This will require further research.

Results for $b_i(\ell)$ and ℓ_s are presented in Sec. S5 as they pertain to ego-level features, the subject of that section.

S5 Individual ego tests

The features captured by $\bar{f}(a,\ell)$ are also shared by $\bar{f}_i(a,\ell)$ for individual egos. This is supported in the main text through the results displayed in Fig. 3, and further tested in other parts of the main manuscript, namely, those that check if the increase of $\bar{f}(a,\ell)$ with ℓ has predictive power such as Figs. 4 and 5. In this section, we complement this evidence by showing primary analyses that allows us to construct the results presented in the main text, as well as additional robustness checks for the main text results.

S5.1 Visual inspection of random sample of $\bar{f}_i$

A simple and illuminating check for the consistency between $\bar{f}_i$ for individual egos and the aggregate result $\bar{f}$ is to plot the series together. Fig. S12 shows the $\bar{f}$ reported in the main text (dark curves), Fig. 1, as well as $\bar{f}_i$ for a random sample of 10 egos in each cohort (light-colored curves). While the results from individual egos are noisier, as expected, the steadiness and monotonicity features are still present at the level of individual egos. Thus, $\bar{f}_i(a,\ell)$ for different egos are generally steady through a large range of values of a, and generally increase with ℓ.

S5.2 Distribution of b_i

The methods discussed in Sec. S4.1 allow us to determine the stable regimes of communication of each $\bar{f}_i(a,\ell)$, along with their associated stable volumes of communication $b_i(\ell)$. Fig. S13 shows the probability distributions of values of b_i for medium and long lifetimes (as defined in Fig. 1 of the main text) of each of the cohorts; the left column of plots shows the results of using the stable region averages and the right column plots show the results from the Mann-Kendall method. The plots also represent the averages of each of the distributions through vertical dotted lines. The color scheme representing lifetime groups is consistent with that of the main text.

The general characteristics of the distributions are very similar over all the plots. First, they show a rapid decay as b_i increases, signaling that in general the values of b_i are distributed over narrow ranges. Second, the long lifetime groups display a slower decay than the medium lifetime groups consistently across all cohorts, in agreement with the monotonic behavior of communication volume with lifetime. Third, both the stable average and Mann-Kendall methods lead to very similar distributions of b_i cohort by cohort, indicating that the results are robust.

For completeness, we also present a version of Fig. 3B of the main text using the Mann-Kendall method (Fig. S14). The figure presented here and the one in the main text have the same qualitative features, including good agreement between the average value of $b_i(\ell)$ and the corresponding $b(\ell)$ for cohort and lifetime ℓ.

S5.3 Distribution of p-values from the Kolmogorov-Smirnov test

As explained in the main text, we study the level of steadiness of $\bar{f}_i(a,\ell)$ as a function of a ego by ego, taking for each time series $\bar{f}_i(a,\ell)$ two parts of equal duration in a around the mid-point of the time series that exclude the first ($a=0$) and last ($a=\lfloor \ell/\Delta a\rfloor \Delta a$) points. The two ranges of elapsed duration ($\Delta a \leq a < \lfloor(1/2)(\lfloor \ell/\Delta a\rfloor - 1)\rfloor \Delta a$ and $\lfloor(1/2)(\lfloor \ell/\Delta a\rfloor - 1)\rfloor \Delta a \leq a \leq \lfloor \ell/\Delta a\rfloor \Delta a - \Delta a$) generate for each ego two samples of $\bar{f}_i(a,\ell)$ at points in a within each of the periods, and we perform a Kolmogorov-Smirnov test to determine if the values of the two samples come from the same distribution. The result of the Kolmogorov-Smirnov test for each ego is a p-value that, the closer it is to 1, the more likely it is that the series $\bar{f}_i(a,\ell)$ is steady. Let us label the p-value obtained for each ego as p_i. We conduct these tests for egos with medium and long lifetimes.

In the main text, we show box plots of the $\{p_i\}_{i\in\mathscr{E}}$ obtained from the tests (Fig 3A) for all cohorts $\mathscr{E}$. Here, we present the probability distributions of these $\{p_i\}_{i\in\mathscr{E}}$ (Fig. S15) over the cohorts and, in addition, the average values of the distributions (vertical dashed lines). The specific averages for each cohort and, respectively, medium and long lifetimes are: for UK 0.84 and 0.73, for IT$_n$ 0.92 and 0.93, for IT 0.84 and 0.86, and for US 0.85 and 0.79. The color scheme is consistent with the main text (Figs. 1 and 3) regarding lifetimes. For reference, we also show with a black dashed line the 0.05 statistical significance level.

As is clearly visible from these results, the distributions concentrate toward $p_i \to 1$ for all cohorts and their averages tend to the same limit, i.e. 1. In the binning used here, a few outlying egos fall under the threshold 0.05. Specifically, for alters with medium lifetimes, the proportion of egos under this threshold of 0.05 are: UK 0%, IT$_n$ 1%, IT 1%, and US 0%. For alters with long lifetimes, the proportions per cohort that fall under the threshold are: UK 4%, IT$_n$ 0%, IT 2%, and US 1%. Thus, the large majority of alters with medium and long lifetimes exhibit steadiness in their communication patterns.

S5.4 Analysis of ℓ_s

The functions $\bar{f}_i(a,\ell)$ do not always stabilize to a flat region. This almost always occurs because ℓ is too small, i.e. when lifetimes are *short* as described in the main text (small fractions of $\bar{f}_i(a,\ell)$ do fail the Kolmogorov-Smirnov test for medium or long lifetimes too, but at rates between 0% and 4% over the different cohorts, thus a negligible effect). As explained in Sec. S4.1, the stable region and Mann-Kendall methods may *fail to converge*, which means they never find a region in which the average slope of $\bar{f}_i(a,\ell)$ is close to 0. On the other hand, when ℓ starts to become large, if a stable region is found, we track the values of the smallest a at which such stable regions begin for each ego. In the methods described in Sec. S4.1, these values are labelled a_m. At the threshold between ℓ being too small, not showing a steady regime, and starting to show stability, a_m and ℓ are very similar as a_M is not too far above a_m. Therefore, as a conservative approximation, we equate a_m to the smallest lifetimes at which $\bar{f}_i(a,\ell)$ for a given ℓ can become stable.

Most $\bar{f}_i(a,\ell)$, as ℓ is increased, eventually exhibit a stable region starting at some $a_m(q_x) = \lfloor q_x/2\rfloor \Delta a$. We collect all such values over egos of a cohort and label them ℓ_s, the minimum lifetime for stable communication. In Fig. S16 we present distributions of ℓ_s for each of the cohorts, the vertical scale is logarithmic and the horizontal scale is linear. The shape of these plots resembles exponential distributions which suggest a narrow set of possible values for ℓ_s.

To provide estimates for the values of ℓ_s at which, generally, $\bar{f}_i(a,\ell)$ becomes steady, we take two approaches. First, we directly calculate the averages of ℓ_s of each of the cohort distributions. These averages can be found in Table S1. We also create a single combined cohort that produces an average ℓ_s of 55.94. A second approach is to assume that the distributions indeed are well approximated by the exponential form $\sim e^{-\ell_s/\upsilon}$, and estimate υ. In turn, υ can be used to provide estimates for ℓ_s. This second approach is well supported by the similarity of the distributions for the different cohorts (Fig. S16B), which suggests that ℓ_s has similar quantitative properties across cohorts. This is a surprising result given the diversity of the egos.

To perform this second approach based on curve fitting we assume $\Pr(\ell_s) = Ce^{-\ell_s/\upsilon}$ where C is the normalization constant. The range of values of ℓ_s can be limited on the left if desired, with a value we call $\ell_{s,\min}$. The average of the distribution requires we determine υ. For this purpose, we use the information in Fig. S16A and perform a least-squares regression of the points using a logarithmic transformation of the vertical axis first but leaving the horizontal scale linear. This gives the equation

$$\log \Pr(\ell_s) = -\frac{1}{\upsilon}\ell_s + \log C \qquad (3)$$

and the slope $1/\upsilon$ is obtained from the least-squares regression. This provides the value $\upsilon \approx 48.7$. Finally, the average of an exponential distribution satisfying $\Pr(\ell_s) = Ce^{-\ell_s/\upsilon}$ with $\ell \geq \ell_s$ is given by $\upsilon + \ell_{s,\min}$. For the combination of cohorts, the smallest ℓ_s is $\ell_{s\min} = 14$, providing a final estimate of 62.7 for average ℓ_s, similar in value to the estimate above based on directly computing the average of $\Pr(\ell_s)$, namely, 55.94.

S6 Information about lifetimes of transient relationships obtained from early communication volume

Figs. 4 and 5 in the main text show that the survival probabilities of alters increases as a function of activity early in the relationship. In this section we provide further evidence of this, by performing robustness checks on those figures. First, we provide justification for the choice of binning used in Figs. 4 and 5. Following that, we show that the behavior seen in Fig. 4 of the main text, which combines the UK, IT, and US cohorts, is also visible in each one of the cohorts separately, including IT_n. To conclude the section, we explore the effect of changing the choices of the window of time in elapsed duration used to assess the relation between early call activity and ultimate lifetime of an ego-alter pair.

S6.1 Overall ego-alter number of calls and exponential binning for γ

For Figs. 4 and 5 of the main text we define g, the communication volume between an ego and alter pair in a specific range of a, selected to be at the initial stages of an observed ego-alter relationship. When we use this parameter to provide estimates for the lifetime ℓ of a relationship, we also group the values of g into exponentially sized bins, labelled by the variable γ. Here we provide the background evidence that such binning choice is necessary given the distribution of numbers of calls seen across ego-alter pairs in our datasets.

Fig S17 shows the probability distribution of the number of calls between each ego-alter pair for the lifetime of the relationship, separated by cohorts (panel A). Panel B shows the distribution of the number of calls fixed to the period of time of relationships with $30 \leq a \leq 60$. As is clearly visible from both panels, the exponential sizes of the bins are well suited to plot the slowly decaying probability distributions, justifying their use in Figs. 4 and 5 of the main text. We find that 3 is a good base for the exponential bins, capturing clearly the distinction between different levels of call activity captured by g.

S6.2 Probability of relationship survival as a function of γ per country

To provide a robustness check for the relationship survival probabilities $P(a \mid a_o, a_f, \gamma)$ presented in the main text, we show the same probability here for each separate cohort (Fig. S18). The results display the same consistent behavior, i.e. that $P(a \mid a_o, a_f, \gamma)$ increases with γ for fixed value of a, indicating that more relationships survive longer the greater the amount of early activity seen.

S6.3 Using different values of a_o and a_f

The definition of g, as indicated above, admits an arbitrarily chosen range of a. Our choice of $a_o = 30$ to $a_f = 60$ in the main text is driven by the fact that this period is long enough (30 days) to capture sufficient activity to provide information about a relationship, and finally is not too long so it remains a *limited* measurement of communication activity rather than the measurement of a large proportion of the activity that, in some sense, one wants to predict.

Fig. S19 shows an extensive check of the effect of varying a_o and a_f on $P(a \mid a_o, a_f, \gamma)$, for the combined cohorts UK, IT, and US; organized in matrix form to study the change systematically. Along the horizontal direction, $a_f - a_o$ is kept fixed while a_o increases, thus exploring the effect of using progressively later windows of observation. Along the vertical direction, $a_f - a_o$ increases while a_o stays fixed.

The first general observation is the remarkable robustness of the behavior of $P(a \mid a_o, a_f, \gamma)$ with respect to a for the variety of choices of a_o, a_f we test. A few additional features emerge worth mentioning. First, if a_o is chosen early in the relationship (say $a_o = 0$), survival curves are closer together and even show inconsistency for the smallest call bin $\gamma = 0$. This is explained by the fact that very early in relationships, communication patterns have not totally settled and therefore longer and shorter lifetime relationships are still somewhat indistinguishable. Second, as $a_f - a_o$ increases, the survival curves separate from each other and call volume g has a greater predictive power. However, a very early choice of a_o dominates over a larger window $a_f - a_o$, indicating the unreliability of trying to predict relationship duration at an extremely early point of the relationship. As can be seen when the choice of a_o becomes 30, reliability returns, even for shorter windows of observation, even down to 15 days. Third, the curves for $\gamma < 2$ appear to be smoother than those for higher values of γ. This occurs due to the number of alters in these bins, as shown in Table S2. Finally, when a_o and $a_f - a_o$ both increase, the curves $P(a \mid a_o, a_f, \gamma)$ separate very widely as functions of γ, signalling the much greater ability of g to predict lifetime. The caveat to this is that, since the purpose of using early call volume within a limited time window is mostly to provide some early estimates of ℓ, it is not practical to increase both a_o and $a_f - a_o$ because in that case the measurement of g in fact amounts to a full measurement of relationship call volume.

S7 Consistency between countries

S7.1 Variations on contour plots

We also test the robustness of the results of Fig. 5 to cohort selection. In that figure of the main text, we combined data from the UK and US to explore how well they predict the Italian cohort. Here, we change our cohort selection in order to test how robust

these results are.

In Figs. S20 and S21 we show two combinations of two countries used to produce the contour plots shown, and the third country is contrasted against those contours. In all cases, as in the main text, the third country's behavior is reasonably predicted. The limited quality in comparison with that of the Fig. 5 in the main text is that the Italian cohort is, by far, the best sampled one leading to a cleaner match of symbols and contours in Fig. 5. Both the UK and US cohorts have limited statistics and therefore produce somewhat coarser results. Nevertheless, their qualitative trends are consistent with those seen in the main text, supporting our conclusions. While other details about the cohorts may play a role, that exploration beyond the scope and goals of the current project.

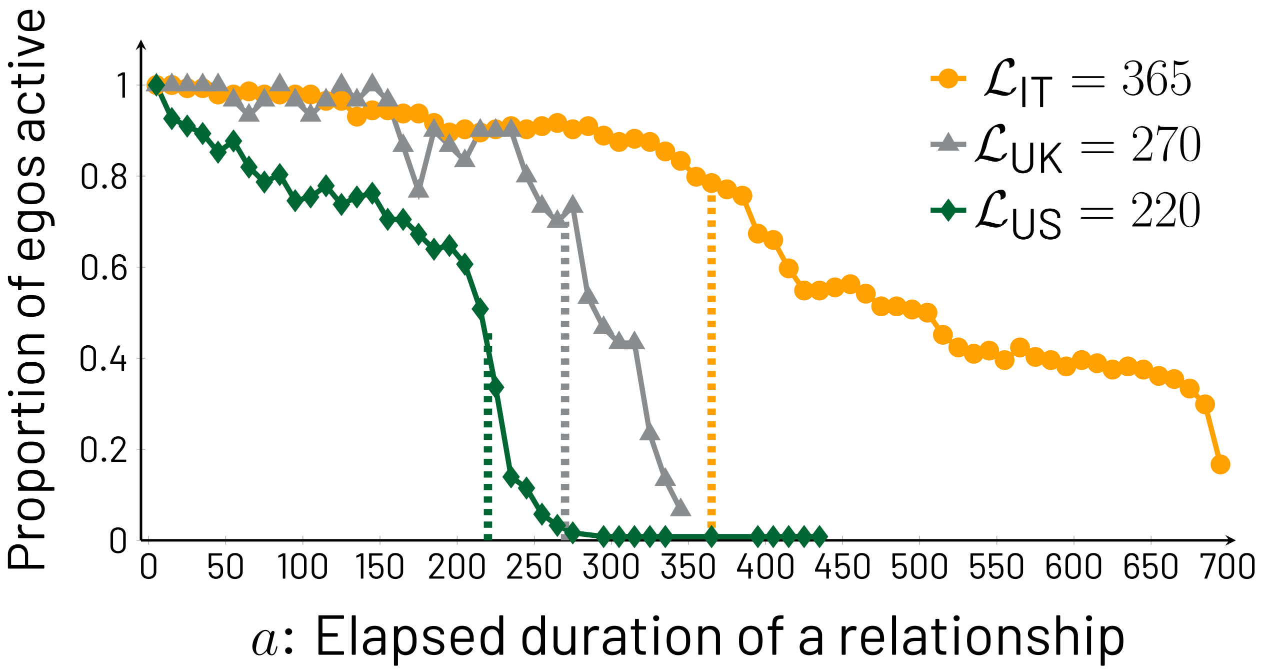

Figure S1. Proportion of egos with at least one active relationship at or above elapsed duration a, country by country. These relationships are only filtered by having a minimum of 3 contacts throughout each data set; in other words, these relationships are not necessarily transient. Vertical dashed lines show the chosen values for $a = \mathscr{L}_{\mathscr{E}}$, for each $\mathscr{E} = \{\mathrm{UK}, \mathrm{IT}, \mathrm{US}\}$. These values are located at the point where the number of egos still having active alters begins to decay rapidly.

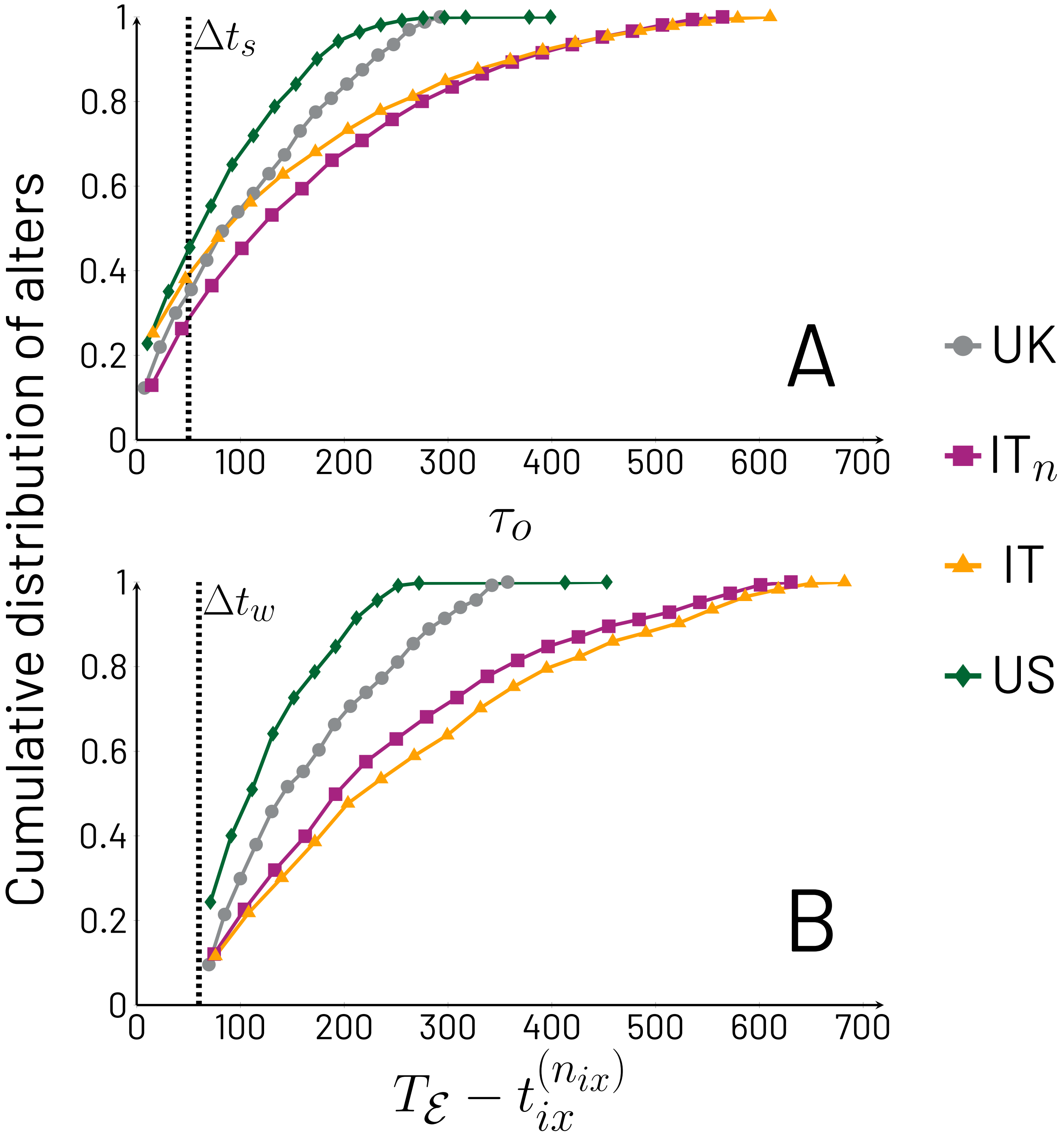

Figure S2. Panel A: cumulative distribution of τ_o, the random variable of the variates $\tau_{o,ix}$ which corresponds to the starting day of relationship ix compared to the beginning of ego x in its respective cohort. These $\tau_{o,ix}$ come from transient relationships. Panel B: cumulative distribution of the number of days between the last phone call between transient ego-alter pair ix and the last day of the cohort, cohort by cohort.

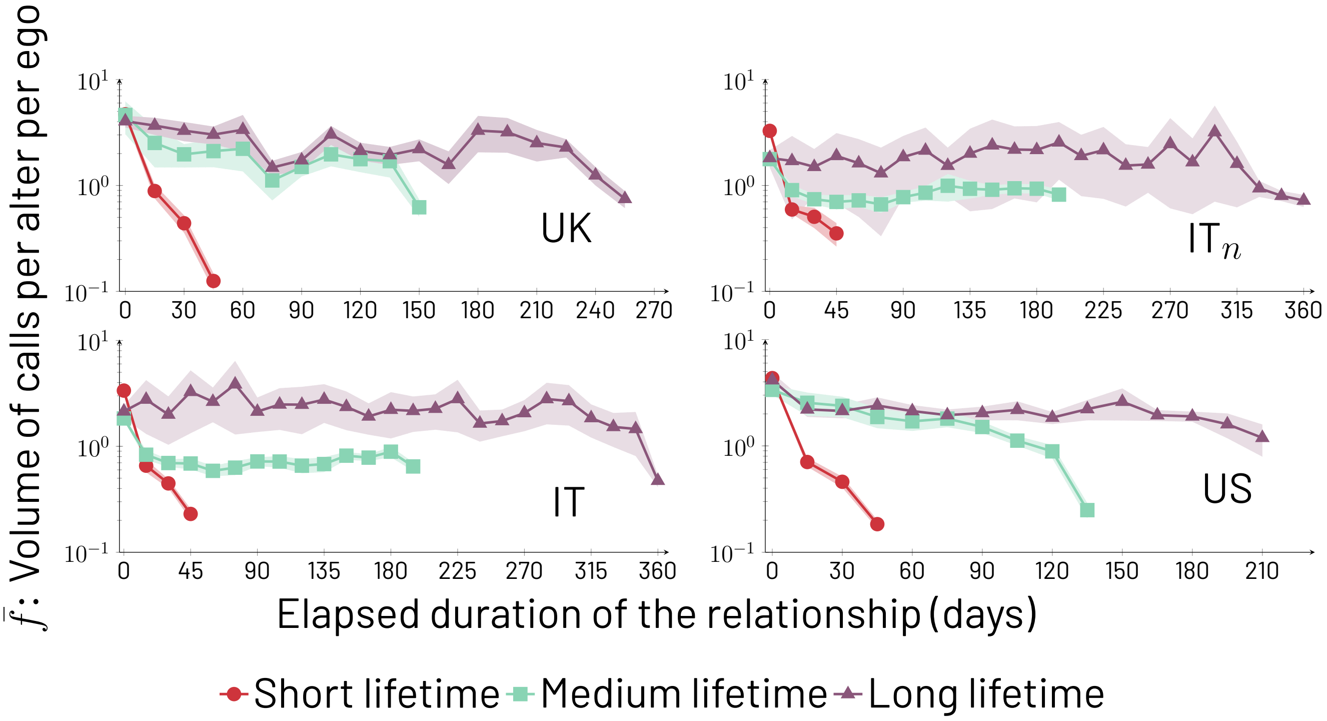

Figure S3. Version of Fig. 1 of the main text with the addition of one standard error (above and below) for each point of $\bar{f}(a,\ell)$, represented as a shaded region with color corresponding to those used for each lifetime group in the paper. All other parameters are the same as those presented in the main text, Fig. 1.

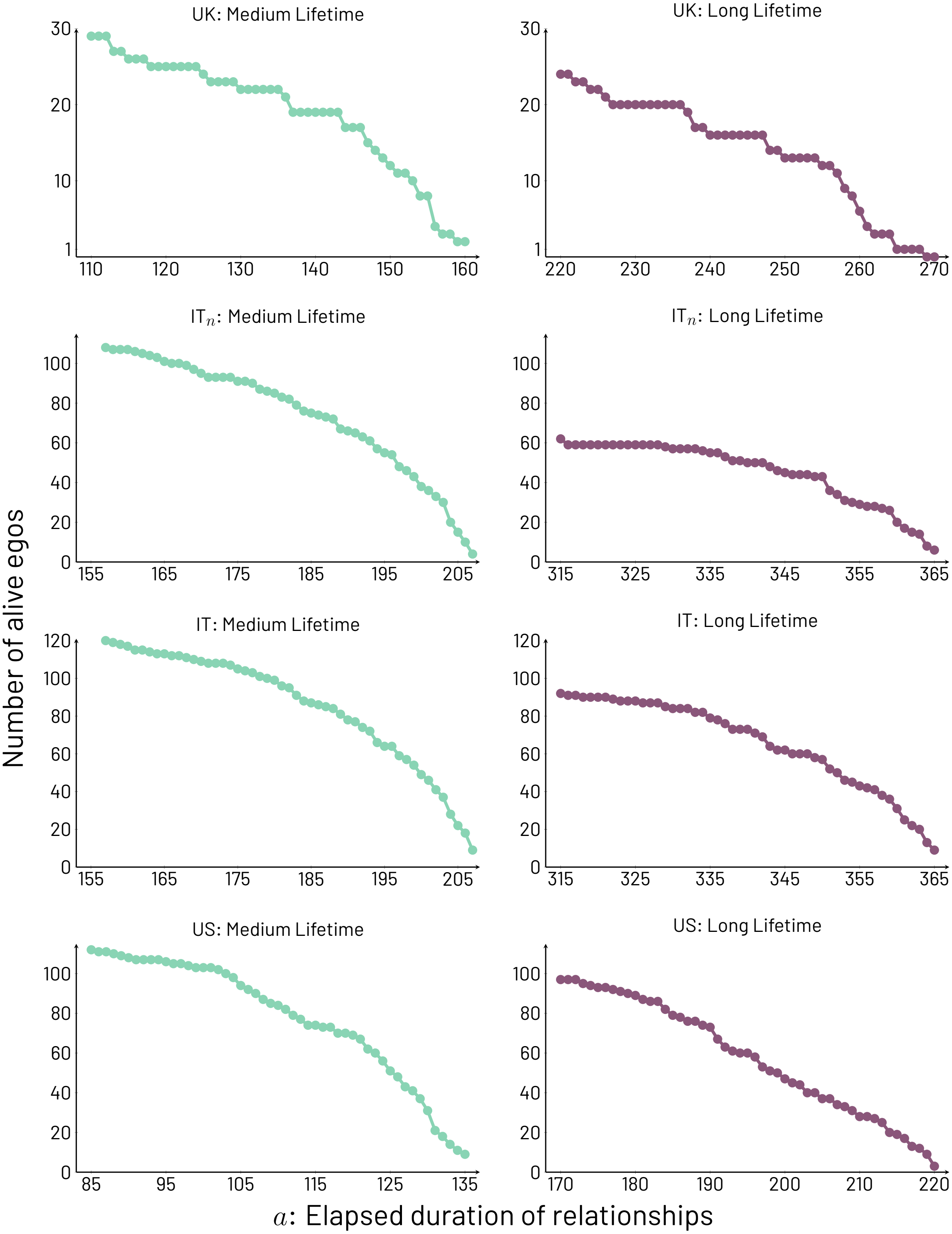

Figure S4. Number of egos with active alters at elapsed durations in the ranges $\ell \leq a \leq \ell + \Delta\ell$ for all countries and lifetime cohorts shown in Fig. 1, main text. Left column shows medium lifetimes, the right columns shows long lifetimes. The colors consistent with the main text, Fig. 1. From these plots, one can observe how the number of active egos decays steadily within the window between ℓ and $\ell + \Delta\ell$, generating the fast decaying effect seen in Fig. 1, a purely statistical effect of the definition of $\bar{f}(a,\ell)$.

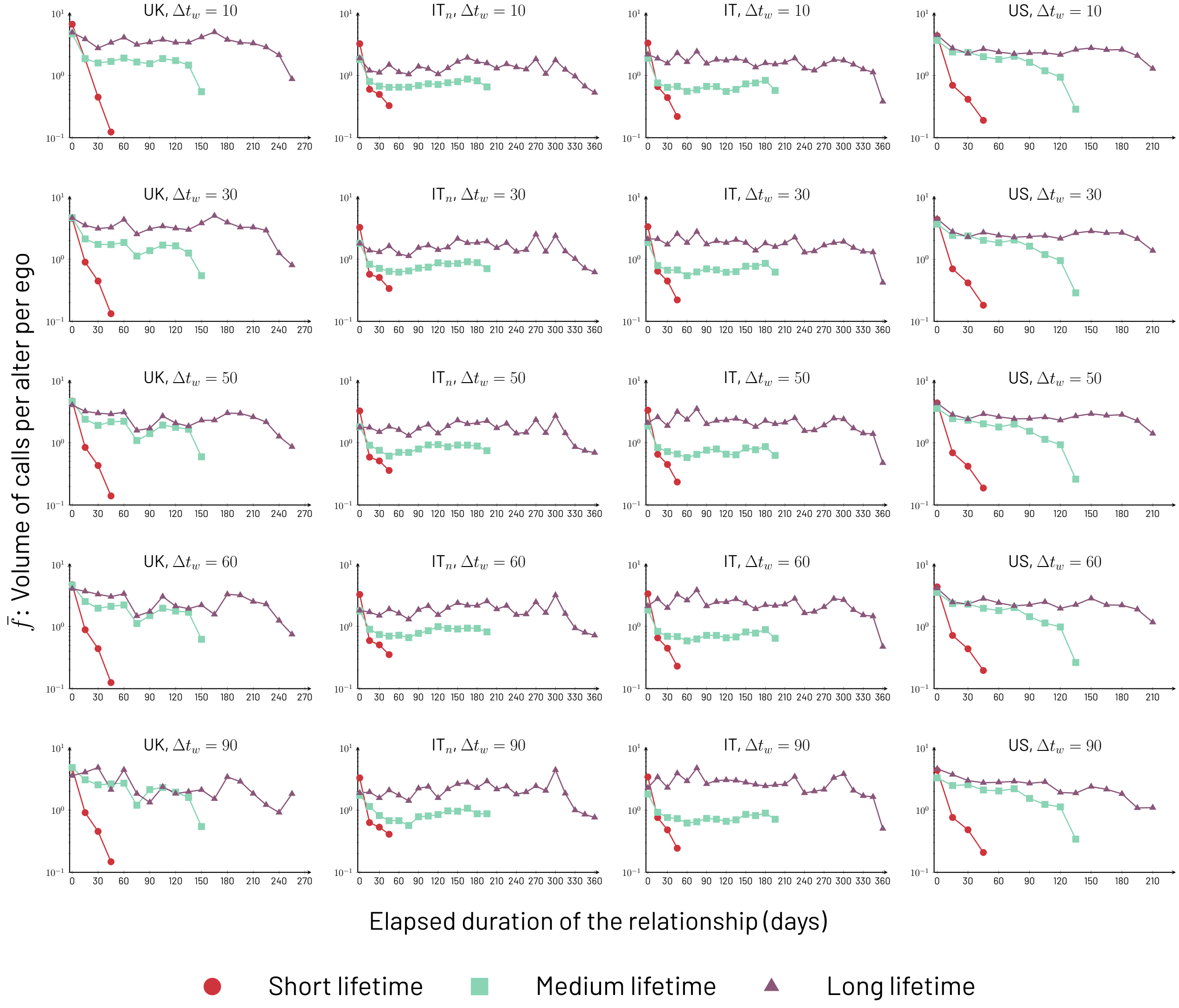

Figure S5. Robustness check of Fig. 1 from the main text with respect to changes in the choice of Δt_w. Each column corresponds to a cohort and each row to a value of Δt_w, all indicated in each plot. The main text uses $\Delta t_w = 60$ days. The qualitative behavior of $\bar{f}(a, \ell)$ is consistent through the choices of Δt_w. Although as Δt_w increases, sampling decreases appreciably and leads to more fluctuations in the signal, the conclusions drawn about Fig. 1 of the main text remain valid.

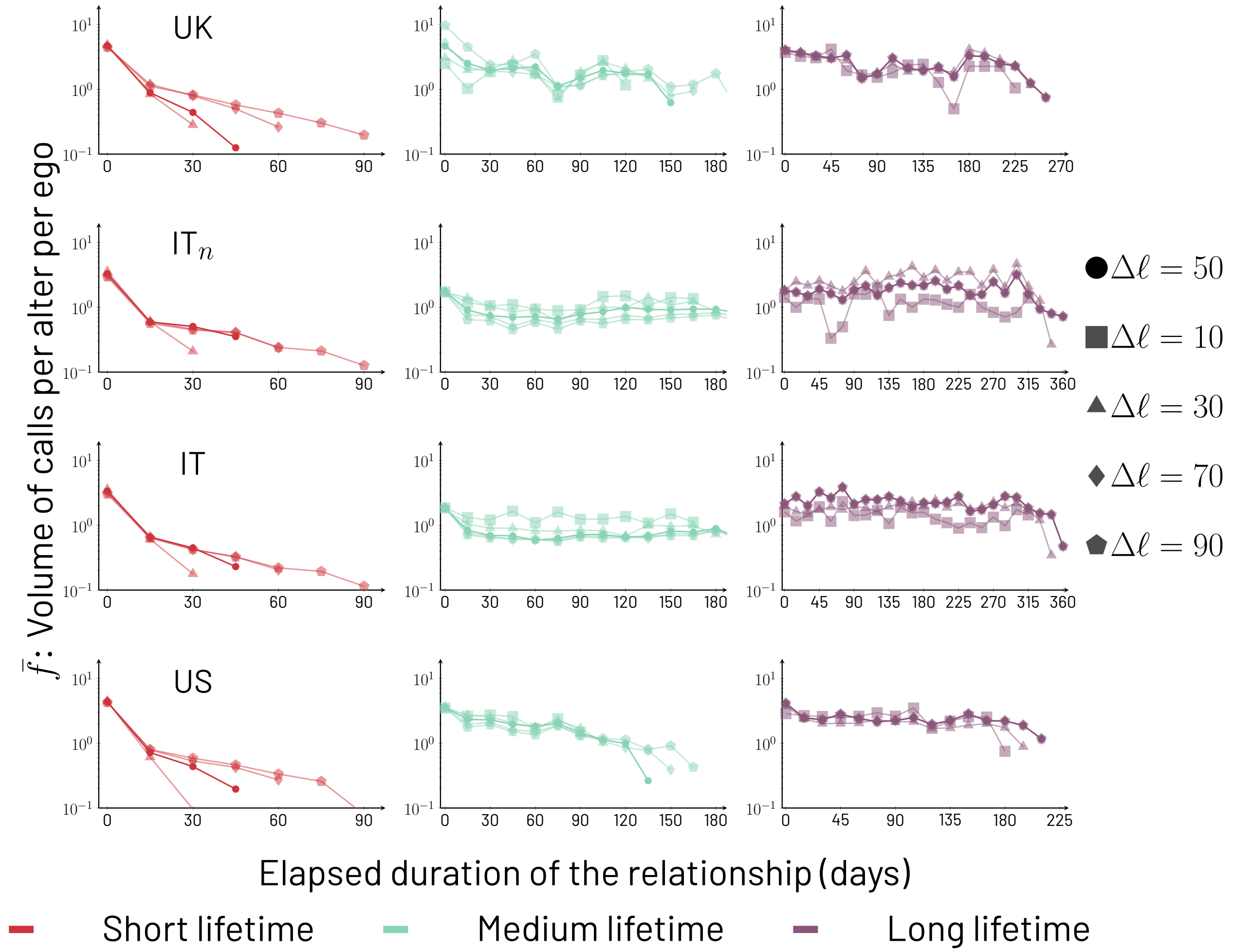

Figure S6. Robustness checks of $\bar{f}(a,\ell)$ with respect to the choice of $\Delta\ell$, separated by lifetime group (short, medium, long). In the main text, $\Delta\ell = 50$ days is used. Each row corresponds to a cohort, and each column to a lifetime group. For small $\Delta\ell$, as expected, the signal fluctuates more. The only qualitative change observed across all the plots is in the column for short lifetimes: as $\Delta\ell$ increases, we observe the gradual emergence of weak plateaus, specially as $\Delta\ell \to \ell_s$, the minimum value at which steadiness emerges for $\bar{f}(a,\ell)$.

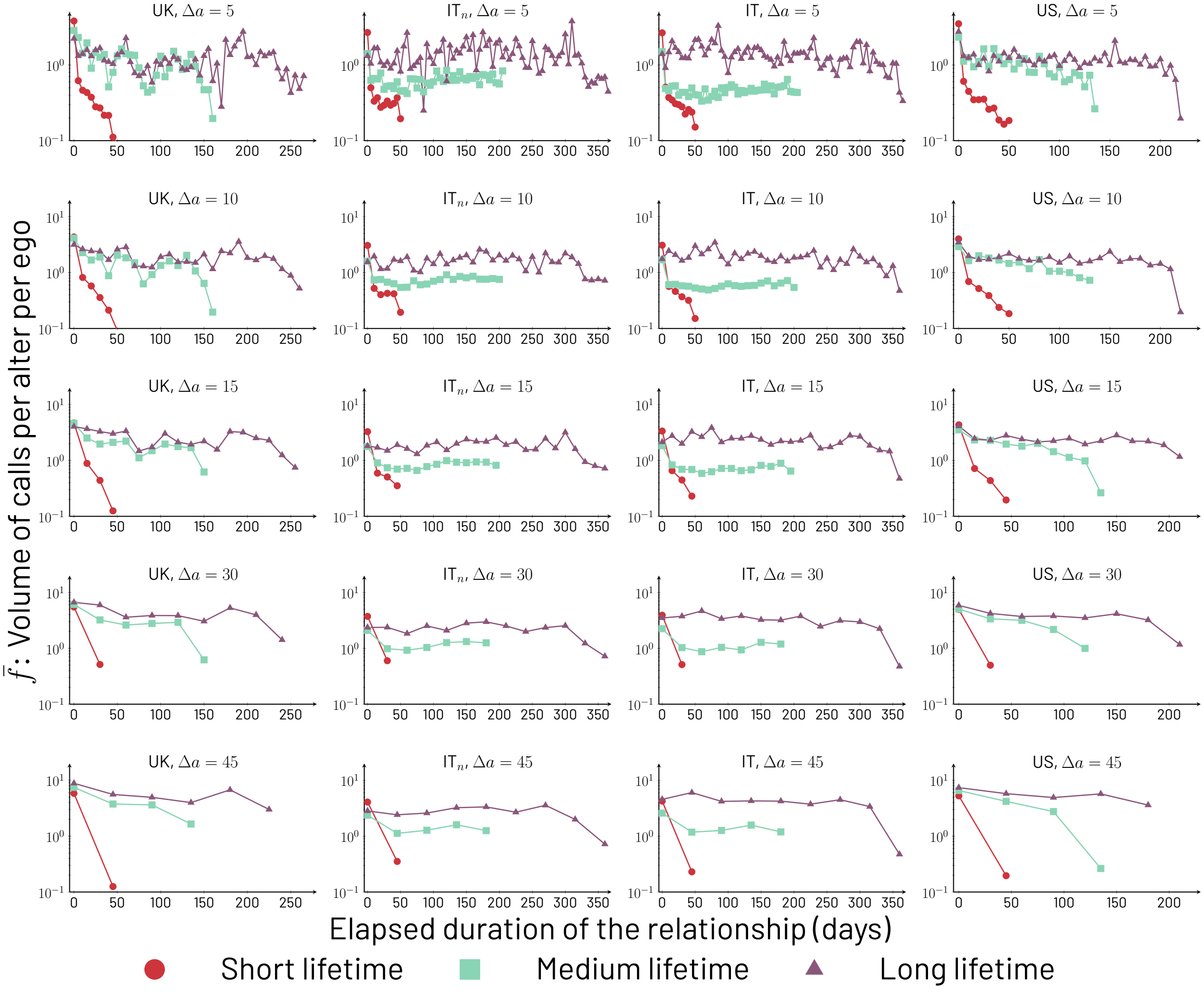

Figure S7. Robustness check of Fig. 1 from the main text with respect to changes in the choice of Δa. Each column corresponds to a cohort and each row to a value of Δa, all indicated in each plot. The main text uses $\Delta a = 15$ days. The qualitative behavior of $\bar{f}(a, \ell)$ is consistent through the choices of Δa. Smaller values of Δa display more fluctuations as expected, but the conclusions drawn about Fig. 1 of the main text remain valid.

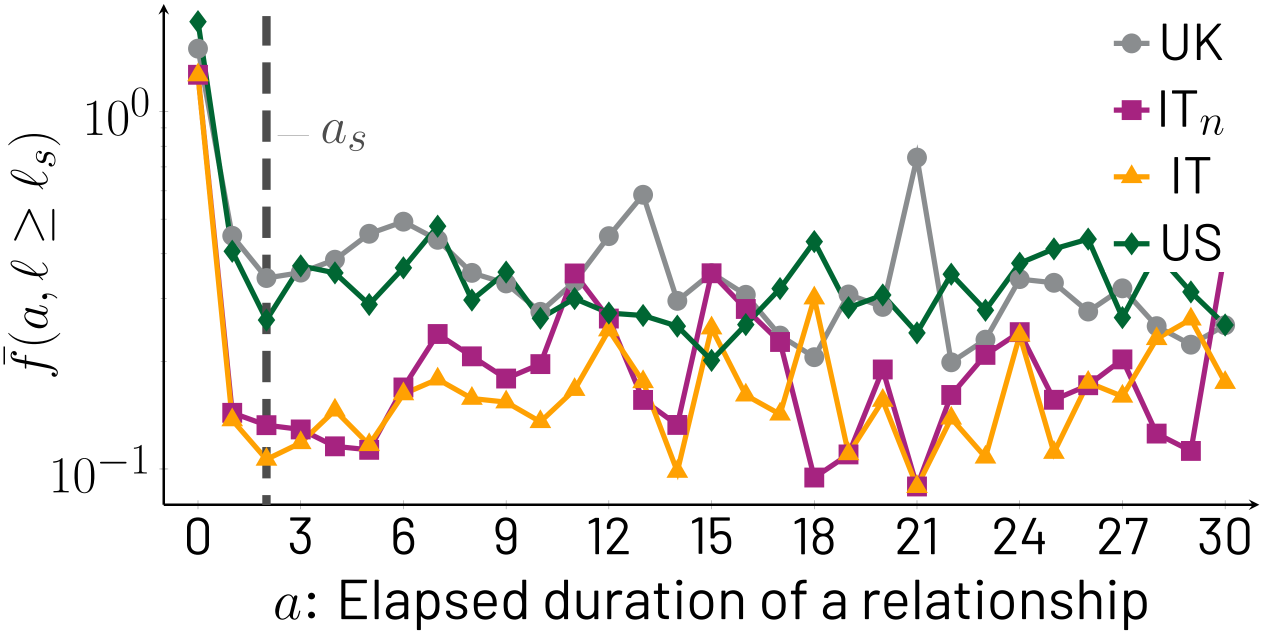

Figure S8. Time series of $\bar{f}(a,\ell \geq \ell_s)$ for all cohorts with $\Delta a = 1$. In all curves, the derivative of $\bar{f}(a,\ell \geq \ell_s)$ is negative for $a \leq 2$. Thus, we identify $a_s = 2$ as the minimum value of a for which Eq. 1 of the main text is valid. For each country, ℓ_s is determined by direct averaging as described in Sec. S5.4

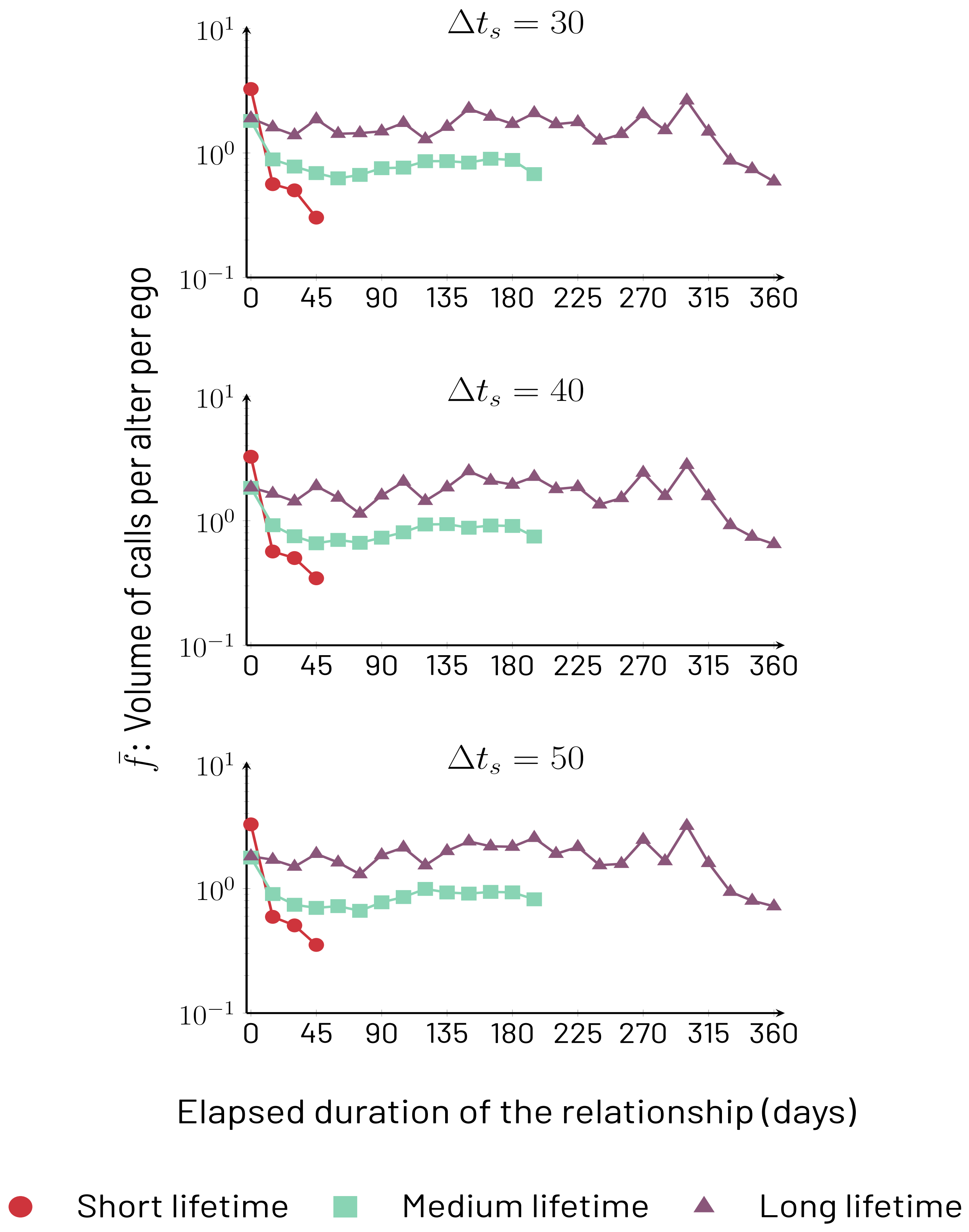

Figure S9. Robustness check for $\bar{f}(a,\ell)$ for different values of Δt_s for the IT_n cohort. The main text shows $\Delta t_s = 50$ days. The results are consistent across the range of values.

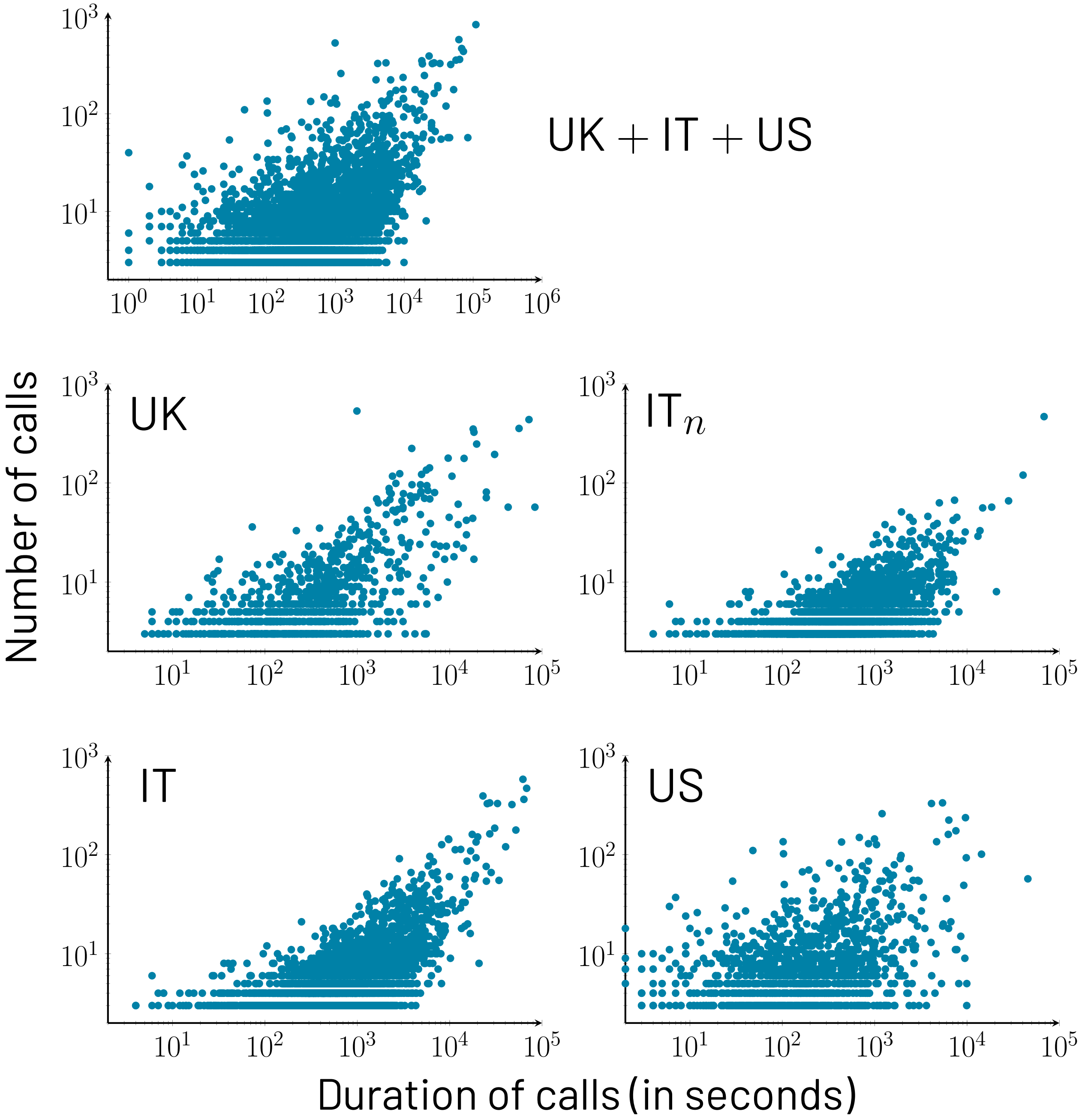

Figure S10. Scatter plots showing the association between the added duration of phone calls in a relationship, and their total number. Results are presented a log-log scale and show a strong positive correlation, with the coefficients presented in the supplementary text.

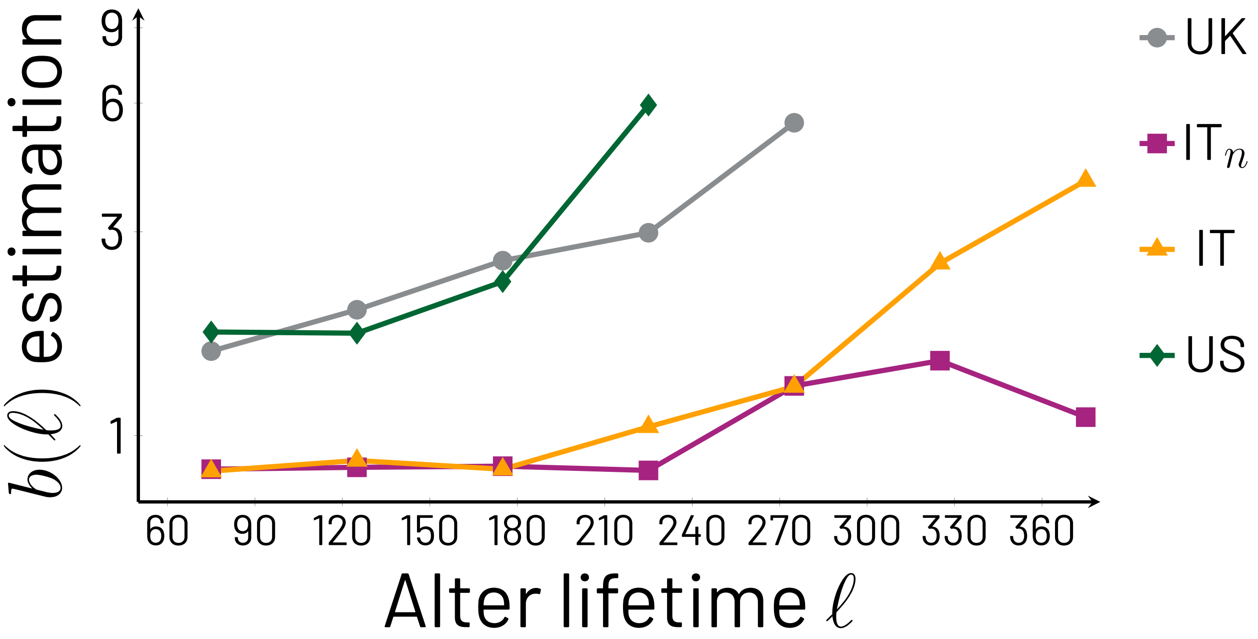

Figure S11. $b(\ell)$ as a function of ℓ obtained through the Mann-Kendall method, cohort by cohort. The vertical axis is in logarithmic scale. Clearly, $b(\ell)$ has an increasing trend with respect to ℓ, with minor exceptions. This result, remarkably similar to Fig. 2 in the main paper, highlights the consistency between the Mann-Kendall and stable region average methods.

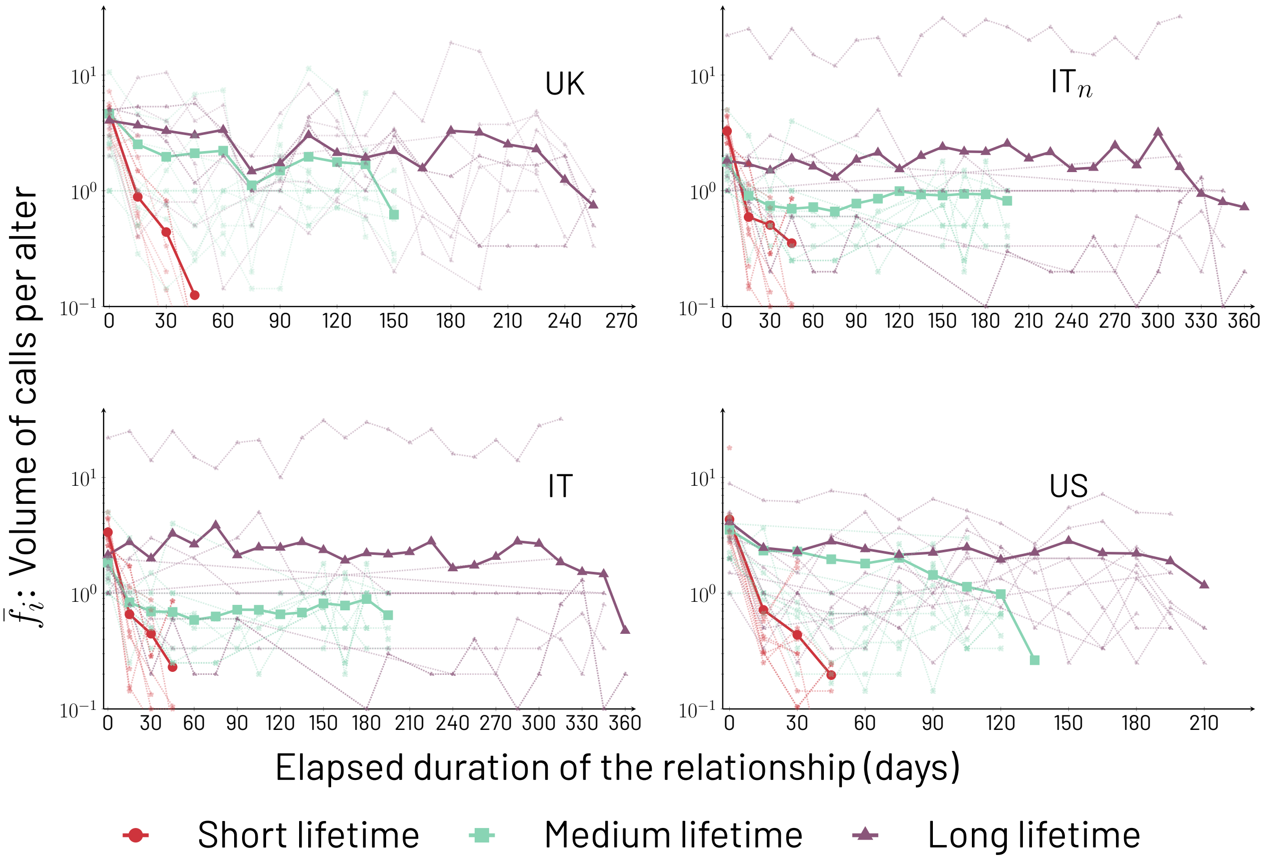

Figure S12. Visual comparison of a sample of $\bar{f}_i(a,\ell)$ curves (light color) with their respective cohort average $\bar{f}(a,\ell)$ (dark color). Cohorts are indicated in each plot. As expected, individual egos exhibit larger fluctuations than their cohort average, yet the fluctuations are generally centered around the averages, providing evidence that the general behavior of individual egos is qualitatively similar to that of the cohort average with respect to monotonicity with respect to ℓ, and steadiness with respect to a. More quantitative evidence for these features being present in the $\bar{f}_i$ is provided by the Kolmogorov-Smirnov test shown in the main text, Fig. 3, as well as below in Fig. S14

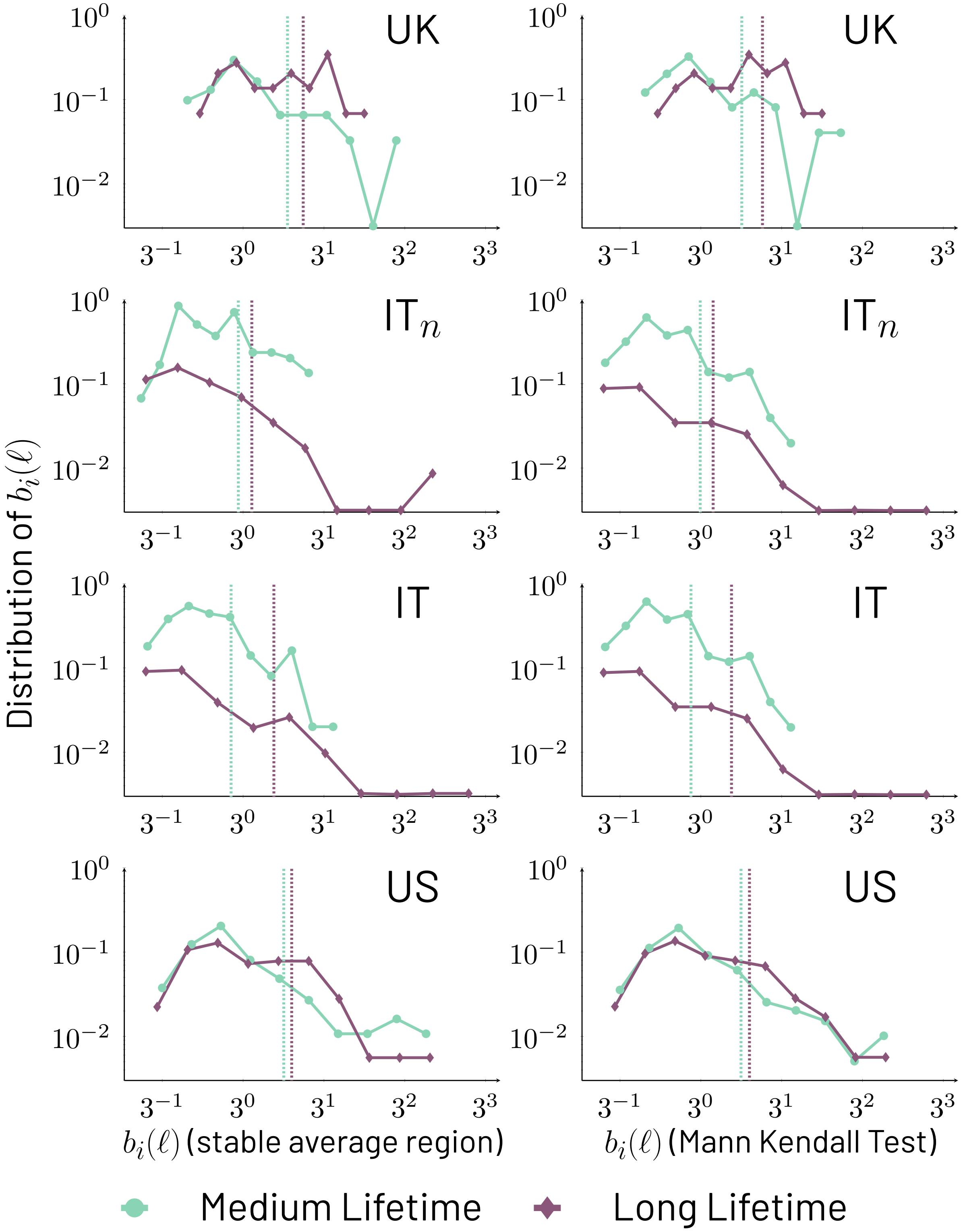

Figure S13. Distribution of $b_i(\ell)$ per cohort (indicated in each plot), with both axes in logarithmic scale. These distributions show that the average stable call volume of egos to their alters does not vary much given a lifetime, supporting the point that $b(\ell)$ and $b_i(\ell)$ are similar given ℓ. In order to show all information in logarithmic scale, all values with frequency 0 are shown with frequency $10^{-2.5}$. Also, since no ego has a value of $b_i(\ell) = 0$, no information was lost due to the scaling of the horizontal axes in any of the plots. All plots to the left use the stable average region method of estimation of $b_i(\ell)$, and the right column shows the estimation using the Mann-Kendall test for trends. The averages of $b_i(\ell)$ over the egos of a cohort are displayed for each cohort and lifetime group with a vertical dashed line.

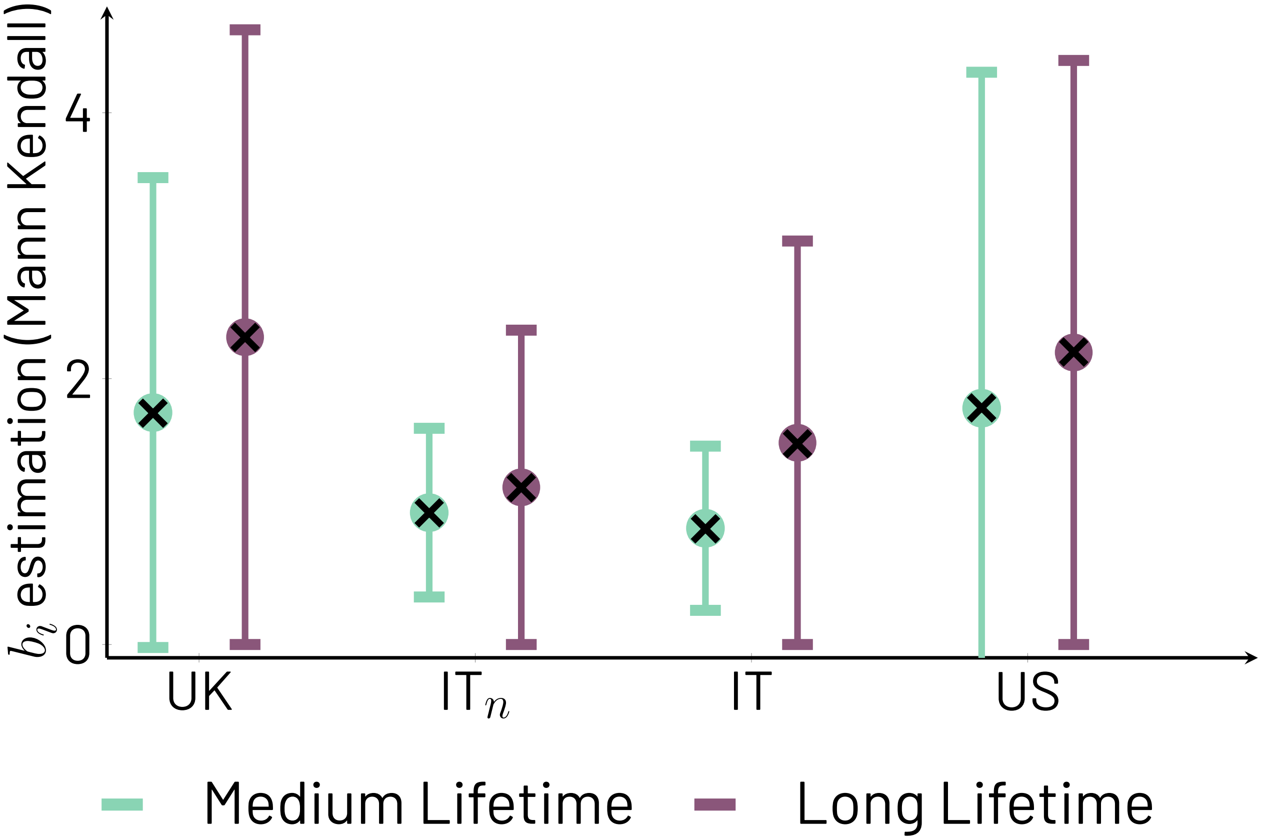

Figure S14. Averages (circles) and standard deviations (lines-whiskers) of $b_i(\ell)$ over egos, determined through the Mann-Kendall method, separated by cohorts and lifetime groups (medium and long). This figure is equivalent to Fig. 3B of the main text, generated with the alternative plateau finding method. The $\times$ symbol represents the value of $b(\ell)$ per cohort and lifetime group. The results support both the point that volume of calls increases with ℓ, and that $b(\ell)$ is a reasonable approximation for the behavior of individual ego patterns of communication with their alters of a given lifetime group.

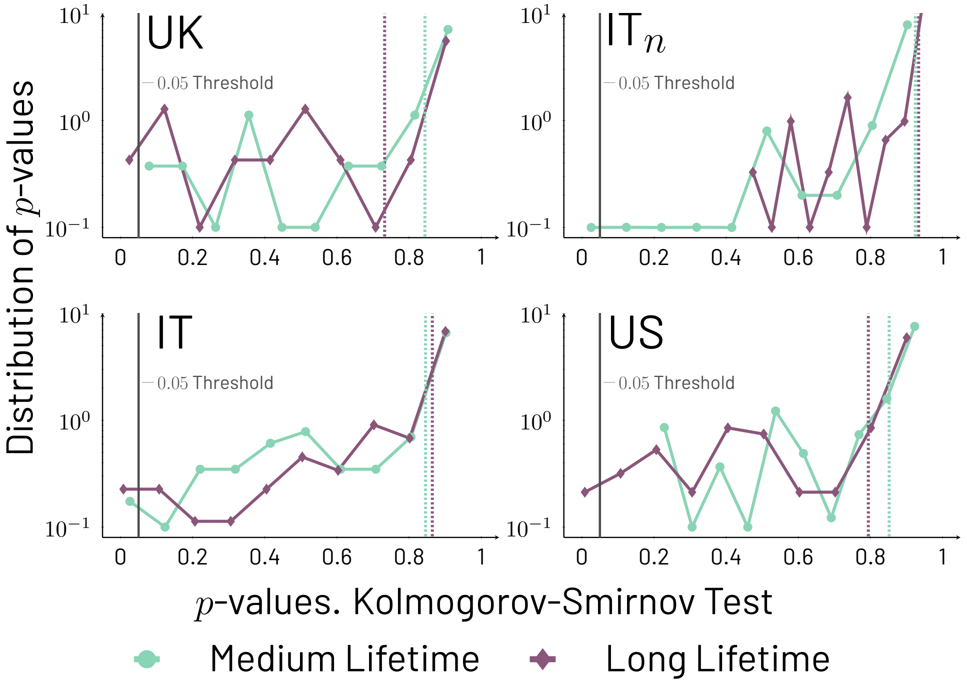

Figure S15. Distribution of p-values obtained from applying the Kolomogorov-Smirnov test to each individual curve $\bar{f}_i$. Each p-value is generated by dividing $\bar{f}_i$ into two equal-sized ranges of a and comparing the values of $\bar{f}_i(a,\ell)$ between the two halves. Medium and long lifetime groups are chosen per cohort and are consistent with those chosen in the main text (cohorts are indicated in the plots). The distributions shown use 10 equal-sized bins for the p-values. Due to the logarithmic scale in the vertical axis of each plot, in order to show all information, values with frequency 0 are shown with frequency 10^{-1}. In all plots, purple and teal dashed lines represent the average of the distribution for alters with medium and long lifetimes, respectively, calculated from the raw data that generates the distributions (values reported in Sec. S5.3). The black vertical line represents $p = 0.05$. The significance of the plots is that they show that the time series $\bar{f}_i(a,\ell)$ for the different cohorts and medium and long lifetimes of Fig. 1 do not have decaying or increasing trends.

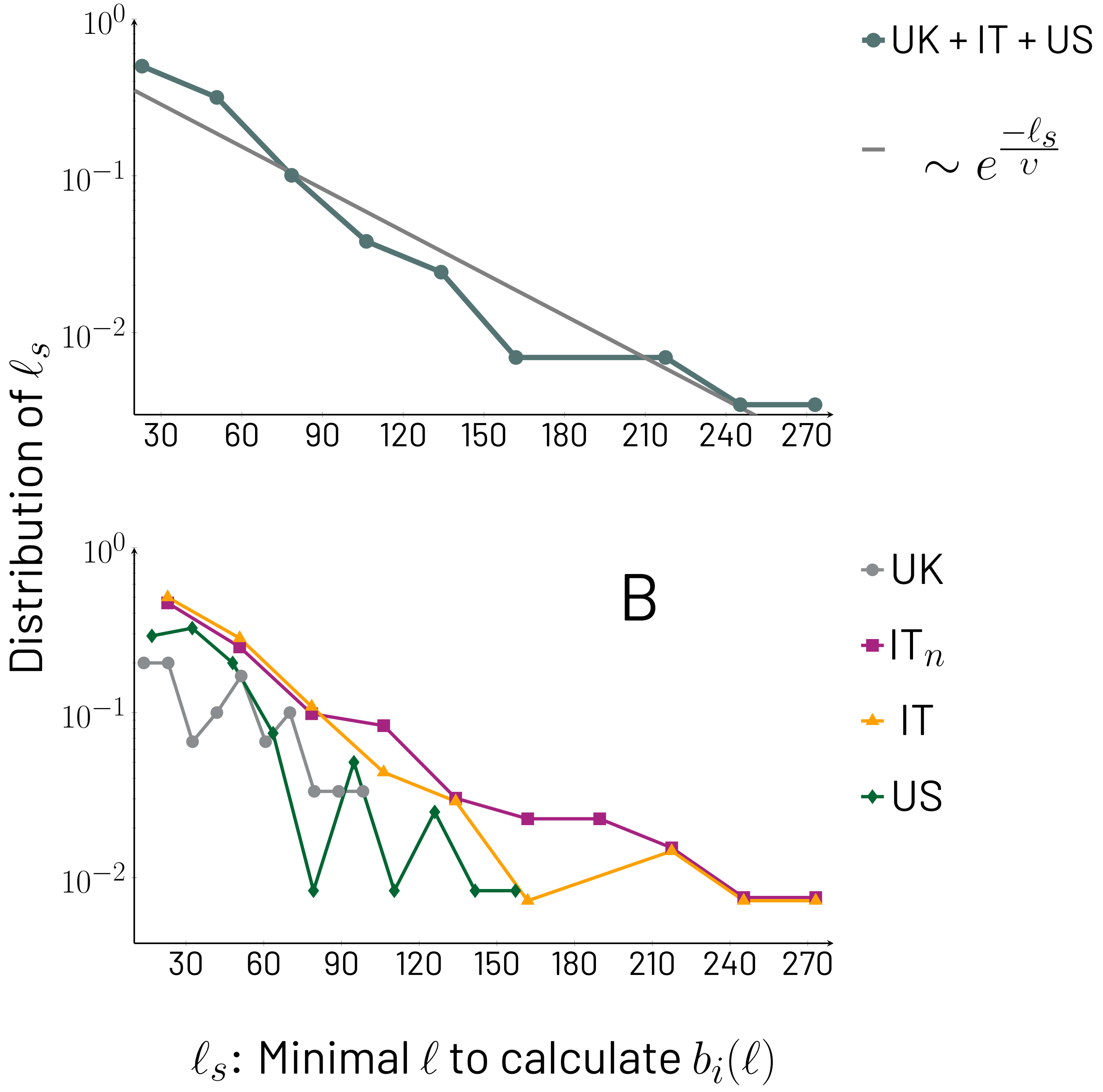

Figure S16. Distribution of ℓ_s plotted with logarithmic vertical scale. Panel A corresponds to the distribution of ℓ_s of all combined ego-alter pairs over all cohorts. The distribution is displayed with symbols, and the line crossing the distribution is the least-squares fit line for Eq. 3. Estimations of the average ℓ_s under the assumption that Eq. 3 is a good approximation as well as the average calculated directly from the data are given within the text of this Supplementary Information and in Tab. S1 (which also contains per cohort averages from panel B of this figure). Panel B shows the distributions of ℓ_s separated by cohort. For the individual cohorts as well as the combined cohort, ℓ_s is obtained for all relationships for which the calculation of $b_i(\ell)$ converges.

Cohort	average ℓ_s		
UK	51.13		
IT_n	66.14		
IT	56.49		
US	56.51		
UK, Italy, and US combined	55.94		
Exponential fit	$\ell_{s,\text{min}}$	υ	Estimated average ℓ_s
UK, Italy, and US combined	14	48.70	62.38

Table S1. Calculation/estimation of average ℓ_s by cohort, all cohorts combined, and exponential fit for all cohorts combined (Eq. 3). When all cohorts are combined, results are obtained from an ordinary least square (OLS) estimation (Fig. S16A). For the individual cohorts as well as the combined cohort, averages are obtained directly from the ℓ_s of all relationships for which the calculation of $b_i(\ell)$ converges. Their distributions are shown in Fig S16B.

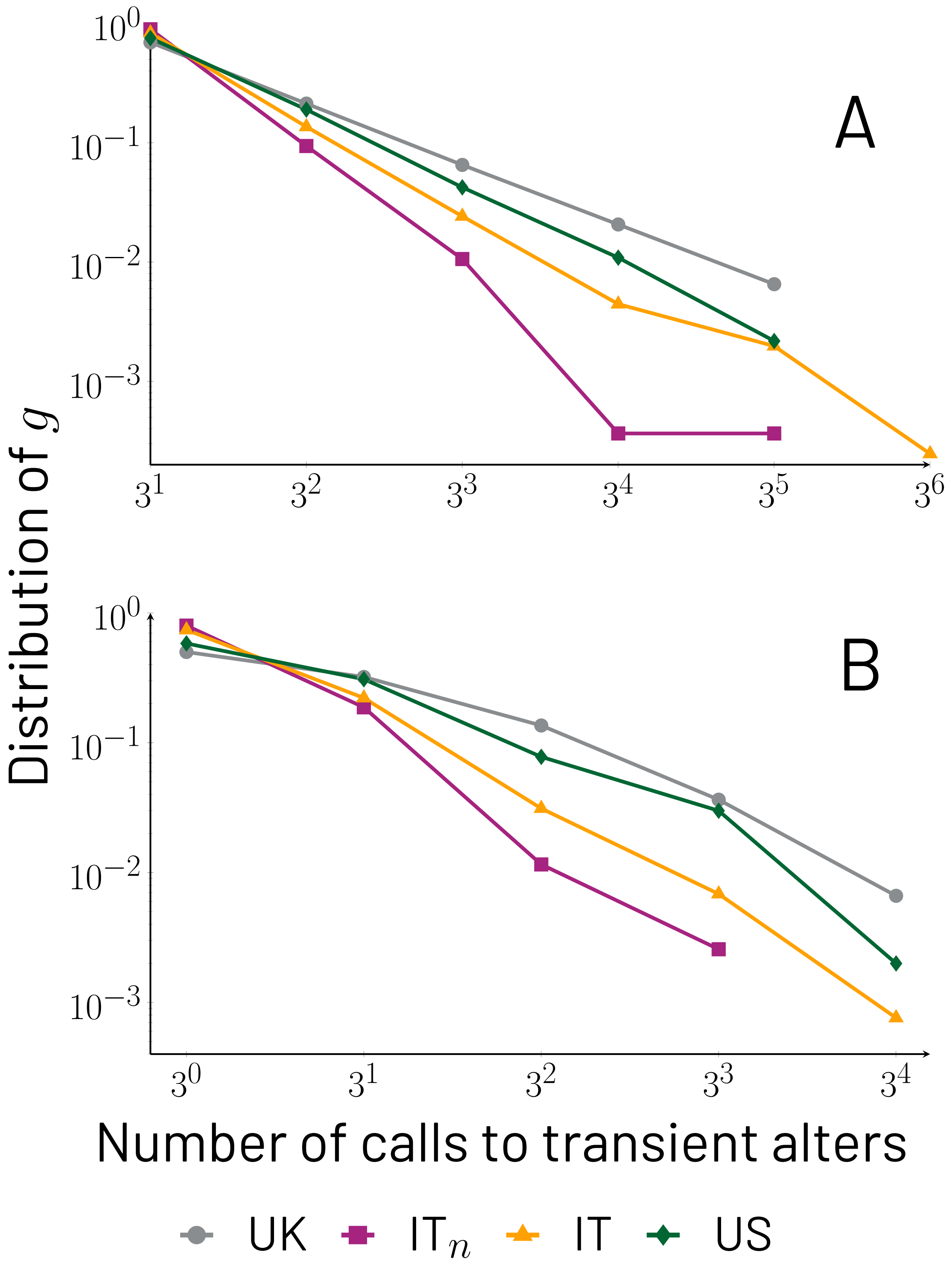

Figure S17. Distribution of g for transient alters, measured between a starting and ending elapsed duration of relationships a_o and a_f. Panel A uses $a_o = 0$ and $a_f = \mathscr{L}_{\mathscr{E}}$ which means all calls for each ego-alter transient relationship with the $\mathscr{L}_{\mathscr{E}}$ is taken into account; Panel B uses $a_o = 30$ and $a_f = 60$, the values used in Figs. 3 and 4 of the main text.

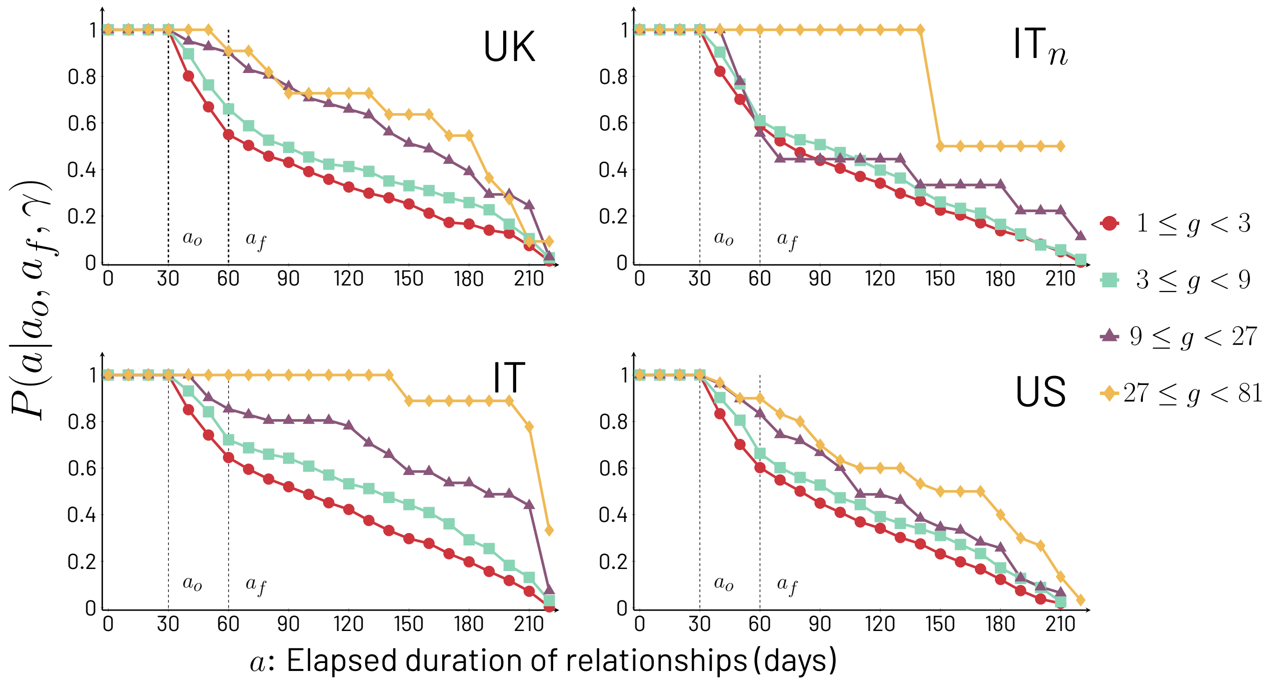

Figure S18. Survival probabilities $P(a \mid a_o, a_f, \gamma)$ of transient alters to duration of at least a for different bins γ of amount of mobile phone calls between $a_o = 30$ and $a_f = 60$ days. Each plot corresponds to a single cohort (indicated in each plot), in contrast to the main text which includes UK, IT, and US. The smaller samples that make up each cohort do lead to noisier results as well as step-wise jumps on the plots. The bins represented by γ as the exponent in $3^\gamma \leq g < 3^{\gamma+1}$ are $\gamma = 0, 1, 2, 3$. As γ increases, and even though the plots display noisier behavior than in Fig. 4 of the main text, the probability of survival also increases in all cohorts, i.e. for $\gamma' > \gamma$, $P(a \mid a_o, a_f, \gamma') > P(a \mid a_o, a_f, \gamma)$, consistent with the conclusions of the main text.

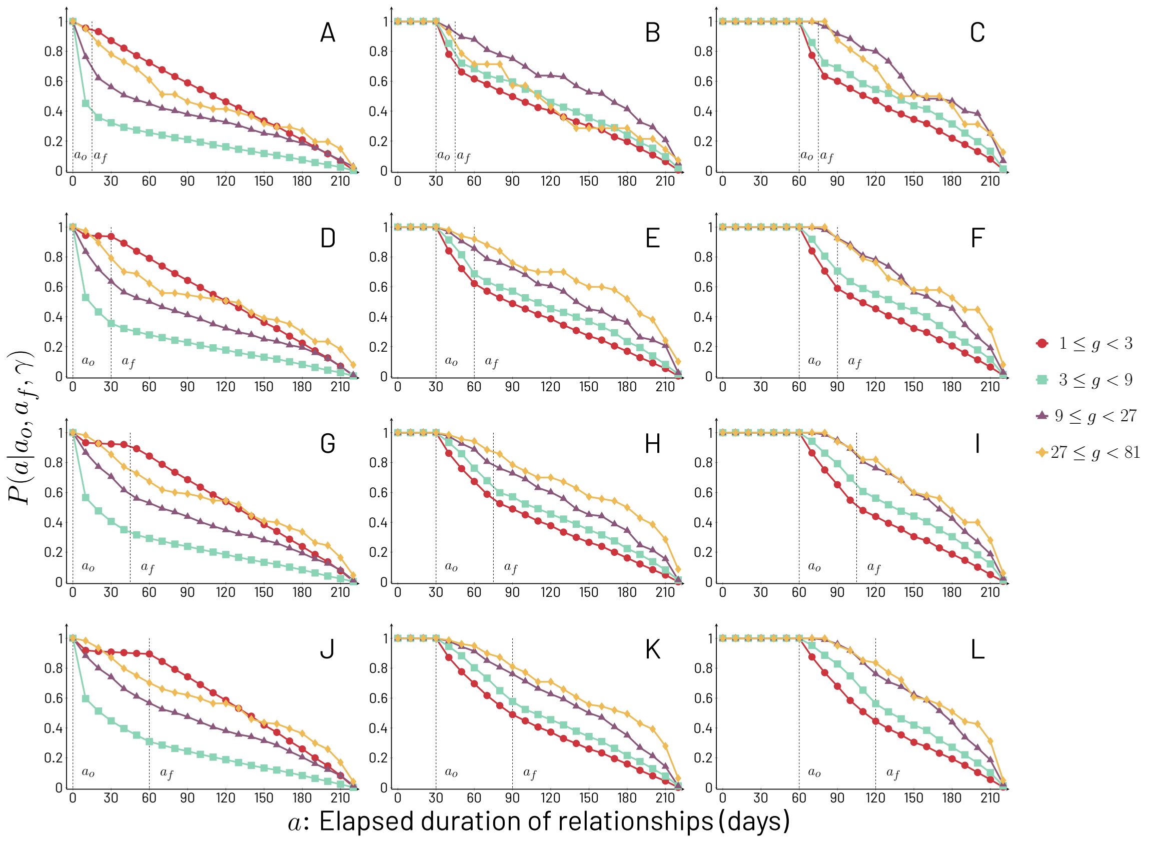

Figure S19. Exploration of survival probabilities $P(a \mid a_o, a_f, \gamma)$ of transient alters to duration of at least a for different bins γ of amount of mobile phone calls between a_o and a_f days. To explore the effects of a_o and a_f, we proceed in a systematic way: each row of plots fixes the value of $a_f - a_o$, while increasing a_o; while each column fixes a_o while increasing $a_f - a_o$. The values of $a_f - a_o$ in order of rows, from top to bottom, are 15, 30, 45, and 60 days; the values of a_o in order of columns, from left to right, are 0, 30, and 60 days. We use the combined data for UK, Italy and US, and therefore, we only look at relationships active for $\ell < \mathscr{L}_{\mathrm{US}} = 220$ days or less, in order to include data for all three cohorts. The bins represented by γ as the exponent in $3^\gamma \le g < 3^{\gamma+1}$ are $\gamma = 0, 1, 2, 3$. The most important effects observed are that if a_o is chosen early in the relationship (say $a_o = 0$), survival curves are closer together and even show inconsistency for the smallest call bin $\gamma = 0$. Also, curves with increasing γ do not separate as broadly. Larger $a_f - a_o$, on the other hand, leads to greater separation between curves of increasing γ, although increasing the window of observation is somehow antithetical to the idea of using g measured in a small time window to predict relationship lifetime. In any case, for reasonable a_o (one that is not too small) we still find, as in the main text, that as γ increases the probability of survival also increases, i.e. for $\gamma' > \gamma$, $P(a \mid a_o, a_f, \gamma') > P(a \mid a_o, a_f, \gamma)$.

γ	A	B	C	D	E	F	G	H	I	J	K	L
0	3820	1186	964	3053	1624	1335	2531	1902	1588	2094	2032	1716
1	3462	381	275	4058	638	500	4425	850	704	4764	1039	857
2	299	107	52	431	151	94	546	183	138	625	228	179
3	38	10	12	72	41	33	102	61	45	113	75	55

Table S2. Number of alters used at each series γ in Fig. S19. Every column corresponds to a panel in Fig. S19. There is a noticeable decrease in the number of alters, as γ increases. Just as for Fig. S19, a combination of the three countries was used for this Table

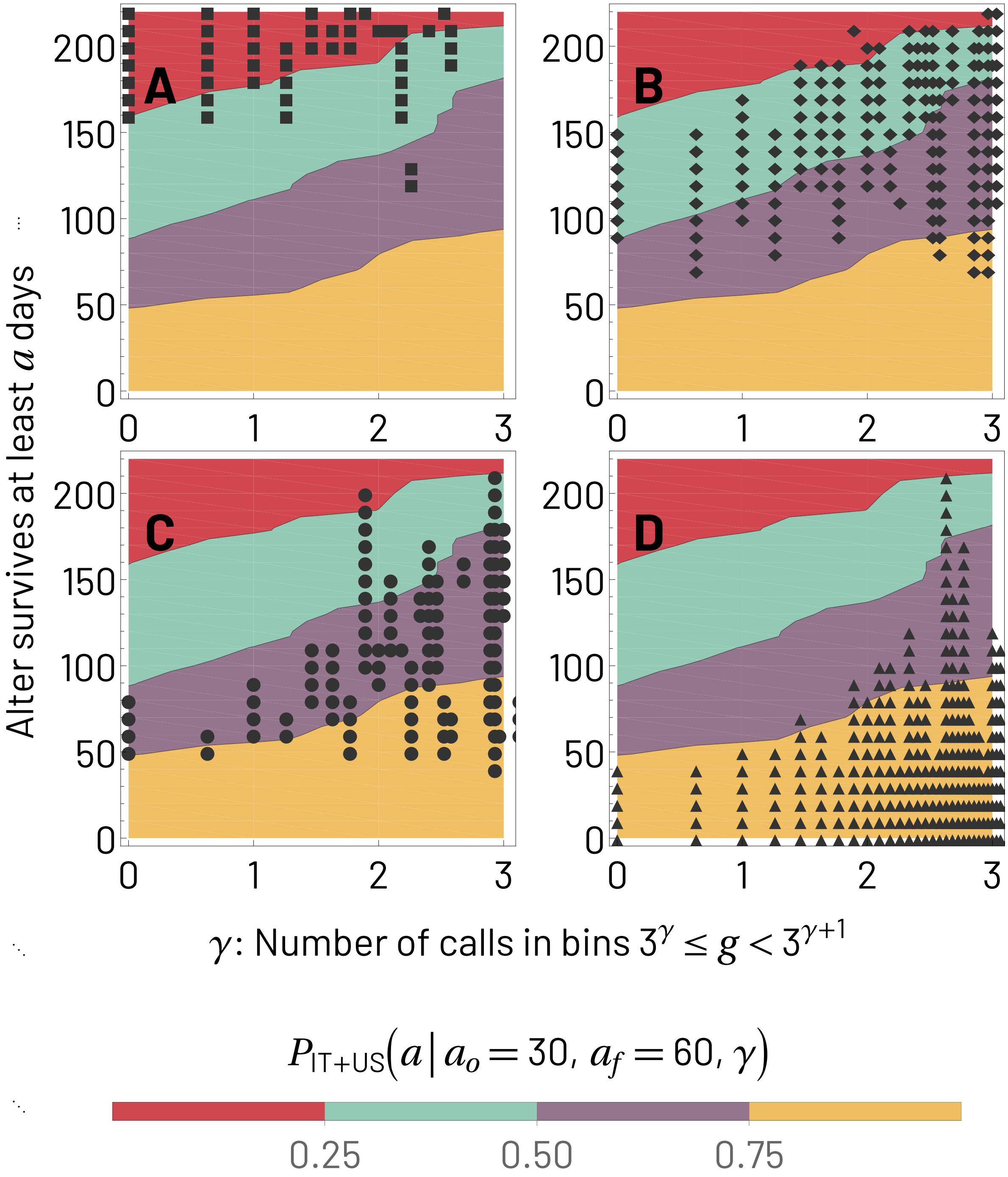

Figure S20. Version of Fig. 5 from the main paper with contours created using US and Italian cohorts and the points correspond to the UK cohort. In the spirit of the main text, the color background represents ranges of $P_{\text{IT+US}}(a \mid a_o, a_f, \gamma)$, namely $[0, 0.25)$ (red), $[0.25, 0.5)$ (teal), $[0.5, 0.75)$ (purple), and $[0.75, 1]$ (yellow). Panel A shows the symbol ■ for $P_{\text{UK}}(a \mid a_o, a_f, \gamma)$ in the interval $[0, 0.25)$, panel B shows the symbol ⋄ for the interval $[0.25, 0.5)$, panel C uses the symbol • for the interval $[0.5, 0.75)$, and panel D uses the symbol ▲ for the interval $[0.75, 1)$ The match in location between the symbols and the colored regions is reasonable throughout values of the probability. However, quality is degraded slightly in comparison to the main text, Fig. 5, as the sample for the UK cohort is considerably smaller than the one for Italy (which forms the symbols of the main text). Overall, the qualitative trend of the results presented is still consistent with those of the main text.

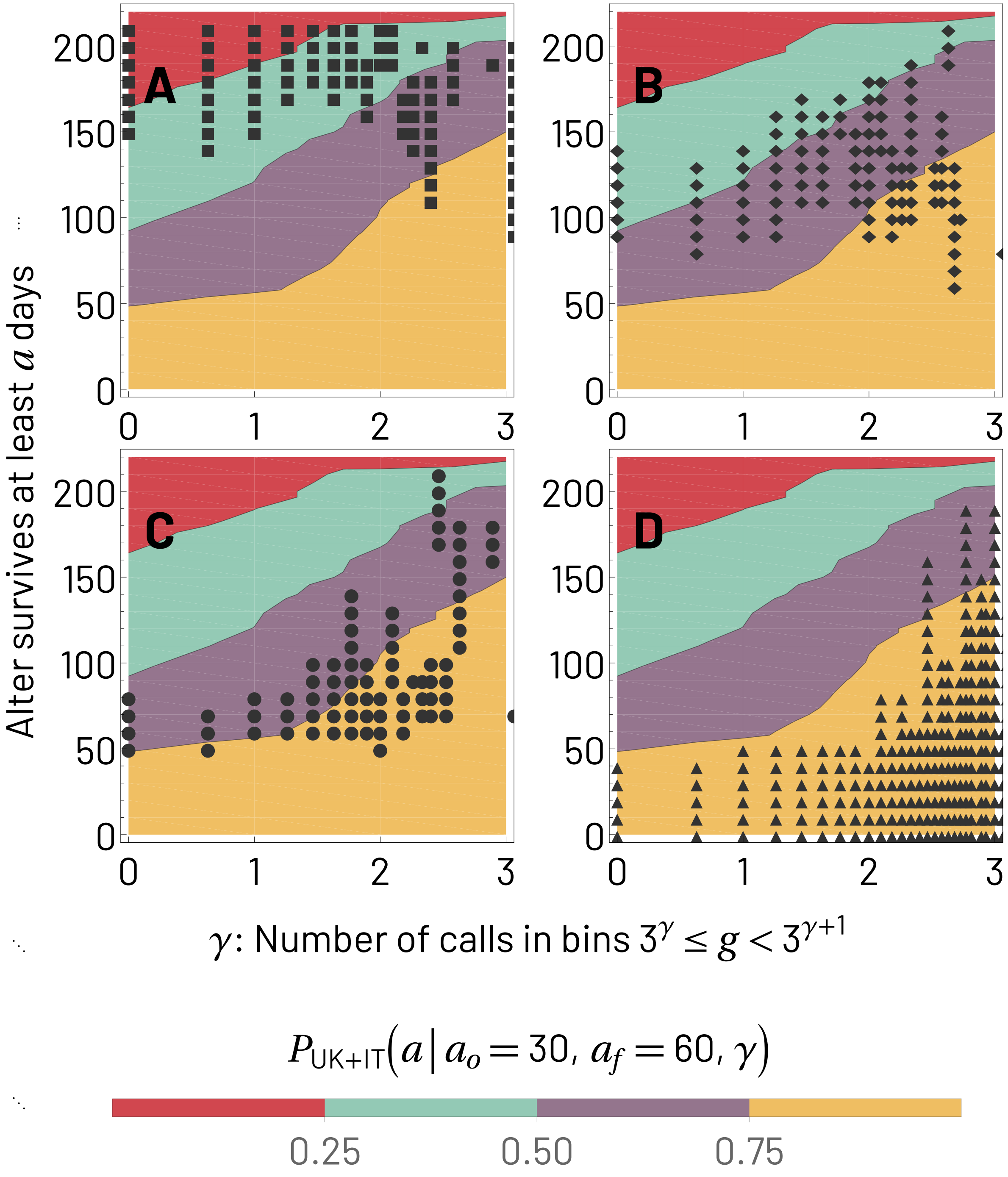

Figure S21. Version of Fig. 5 from the main paper with contours created using UK and Italian cohorts and the symbols correspond to the US cohort. In the spirit of the main text, the color background represents ranges of $P_{\text{UK+IT}}(a \mid a_o, a_f, \gamma)$, namely $[0, 0.25)$ (red), $[0.25, 0.5)$ (teal), $[0.5, 0.75)$ (purple), and $[0.75, 1]$ (yellow). Panel A shows the symbol ■ for $P_{\text{US}}(a \mid a_o, a_f, \gamma)$ in the interval $[0, 0.25)$, panel B shows the symbol ◇ for the interval $[0.25, 0.5)$, panel C uses the symbol • for the interval $[0.5, 0.75)$, and panel D uses the symbol ▲ for the interval $[0.75, 1)$. The match in location between the symbols and the colored regions is best achieved for largest probabilities, i.e. dark yellow and purple regions. For lower probability regions, the match is not as good although it has the correct trend of dependence of survival with respect to g, namely, more calling means longer survival. The smaller size of the US cohort plays a role. Overall, the qualitative trend of the results presented is still consistent with those of the main text.

References

1. Roberts, S. G. & Dunbar, R. I. The costs of family and friends: an 18-month longitudinal study of relationship maintenance and decay. *Evol. Hum. Behav.* **32**, 186–197, DOI: 10.1016/j.evolhumbehav.2010.08.005 (2011).

2. Aharony, N., Pan, W., Ip, C., Khayal, I. & Pentland, A. Social fmri: Investigating and shaping social mechanisms in the real world. *Pervasive Mob. Comput.* **7**, 643–659 (2011).

3. Centellegher, S. *et al.* The mobile territorial lab: a multilayered and dynamic view on parents' daily lives. *EPJ Data Sci.* **5**, 1–19 (2016).

4. Saramäki, J. *et al.* Persistence of social signatures in human communication. *Proc. Natl. Acad. Sci.* **111**, 942–947 (2014).

5. Kendall, M. *Rank Correlation Methods, p 160* (Charles Griffin, London, 1955).

6. Mann, H. B. Nonparametric tests against trend. *Econom. J. econometric society* 245–259 (1945).

7. Hamed, K. H. & Rao, A. R. A modified mann-kendall trend test for autocorrelated data. *J. Hydrol.* **204**, 182–196, DOI: 10.1016/s0022-1694(97)00125-x (1998).

8. Hussain, M. & Mahmud, I. pymannkendall: a python package for non parametric mann kendall family of trend tests. *J. Open Source Softw.* **4**, 1556, DOI: 10.21105/joss.01556 (2019).
